# A WENO-solver combined with adaptive momentum discretization for the Wigner transport equation and its application to resonant tunneling diodes

**DOI:** 10.1016/j.jcp.2014.12.026

**Published:** 2015-03-01

**Authors:** Antonius Dorda, Ferdinand Schürrer

**Affiliations:** Institute of Theoretical and Computational Physics, Graz University of Technology, 8010 Graz, Austria

**Keywords:** Wigner function approach, Quantum transport, Resonant tunneling diode, Weighted essentially non-oscillatory scheme

## Abstract

We present a novel numerical scheme for the deterministic solution of the Wigner transport equation, especially suited to deal with situations in which strong quantum effects are present. The unique feature of the algorithm is the expansion of the Wigner function in local basis functions, similar to finite element or finite volume methods. This procedure yields a discretization of the pseudo-differential operator that conserves the particle density on arbitrarily chosen grids. The high flexibility in refining the grid spacing together with the weighted essentially non-oscillatory (WENO) scheme for the advection term allows for an accurate and well-resolved simulation of the phase space dynamics. A resonant tunneling diode is considered as test case and a detailed convergence study is given by comparing the results to a non-equilibrium Green's functions calculation. The impact of the considered domain size and of the grid spacing is analyzed. The obtained convergence of the results towards a quasi-exact agreement of the steady state Wigner and Green's functions computations demonstrates the accuracy of the scheme, as well as the high flexibility to adjust to different physical situations.

## 1 Introduction

Nowadays, well-established approaches for the simulation of charge transport in semiconductor devices are based on drift-diffusion and hydrodynamic models. Such macroscopic descriptions enable a rather quick computation and therefore, an industrial application. But, owing to the continuous progress made in device fabrication, microscopic descriptions of charge transport become of prime importance. One approach of this kind is to make use of the semi-classical Boltzmann transport equation (BTE) [1,2]. Simulations based on the BTE have attained great success in many different applications, but fail, as soon as quantum mechanical effects dominate the device behavior. A prototypical example for such a device is a resonant tunneling diode (RTD) [3,4]. In this case, a description of the charge carriers as localized particles becomes invalid and a fully quantum mechanical treatment is needed instead. In order to allow for a realistic device simulation, it is essential to employ methods which enable the description of open quantum systems where elastic and inelastic scattering mechanisms are present in addition. Two of the most prevalent approaches of this kind are the non-equilibrium Green's functions (NEGF) [3,5] and the Wigner transport equation (WTE) [4,6,7]. In this work, we will focus especially on a deterministic solution of the latter one. The Wigner function shares many analogies with its classical counterpart, but with the essential difference, that the function is in general not positive definite. Since its marginal distributions are proper probability distributions and since measurable quantities are calculated in the same manner as in the case of the Boltzmann phase space distribution, the Wigner function is commonly termed quasi-distribution as well.

The application of the WTE to the simulation of charge transport has been investigated by many groups in the past decades. Following the pioneering work of Frensley [8], extensions of the basic formalism by including a spatially dependent effective mass [9], by coupling the WTE to Poisson's or Schrödinger's equation [10,11], and by including scattering mechanisms have proven to enable a realistic device simulation [7,12–15]. The solution strategies employed for the WTE are twofold: On the one hand, Monte Carlo schemes have been devised and on the other hand, various discretizations of the WTE for a deterministic solution exist. The Monte Carlo (MC) approaches are either based on the concept of particle affinity [16] or on the concept of particle sign [17]. In particular, the second method has shown to be successful in tackling multi-dimensional [18] and many-body [19] quantum problems. In general, a close agreement with NEGF computations could be achieved and for an introduction to the MC-method we refer to [4,7]. In the case of a deterministic solution of the WTE, the treatment of the pseudo-differential operator, which accounts for the non-local action of the potential, is much more involved than that of terms in classical transport equations. The most common strategy is to make use of the fact that the eigenfunctions of the pseudo-differential operator are plane waves, so that a Fourier transformation allows for an efficient evaluation. The superior scaling of the fast Fourier transform [20] is also made use of in the spectral methods developed in [21] or [22]. The second term in the WTE coincides in the parabolic band approximation with the advection term (also called diffusion term) in the BTE. Well-developed discretization schemes from classical transport theory can thus be employed. In [11,12,23] various higher order finite difference stencils have been applied together with a convergence analysis. The adaptive, high-order scheme in [22] makes use of Gauss–Lobatto collocation points for the advection term.

In this work, we employ a weighted essentially non-oscillatory (WENO) [24] scheme for the advection term, well known from applications within the BTE [2,25,26]. The scheme allows for a highly accurate estimation of the flux in smooth regions of the underlying function, and at the same time, avoids the creation of spurious oscillations at discontinuities or regions with large gradients. Regarding the pseudo-differential operator, even though the fast Fourier transform allows for an efficient evaluation in order $N_{p}\log N_{p}$ steps, certain restrictions are associated with it. On the one hand, the $N_{p}$ points for the momentum grid have to be chosen equidistantly and on the other hand, in order to be consistent with the continuity equation, the momentum grid and the spatial grid are interrelated and the discretizations of each one cannot be chosen independently. From our point of view, these restrictions may cause difficulties to resolve the Wigner function properly especially in situations where strong quantum effects are present, such as in the case of resonant tunneling devices. We thus propose a novel numerical scheme which allows for a highly flexible choice of the grid in order to adjust to different physical situations. The scheme follows similar ideas as in finite element or also finite volume methods, namely to approximate the Wigner function locally by piecewise polynomials. It is ensured by construction that the discretized WTE is consistent with the continuity equation without further conditions, so that the particle density is conserved for arbitrarily chosen grids. The local approximation enables one to change the resolution in different regions of the phase space by orders of magnitude. This allows us to properly resolve all details of the steady state Wigner function of a RTD, consisting of a rather smooth shape on which very short-scaled and large-valued oscillations are superimposed in certain regions of the phase space.

As a test case for the method we consider the simplest model of a RTD, given by a one-dimensional, static potential with a homogeneous doping profile and a constant effective mass. In addition, scattering mechanisms are neglected and fully coherent transport is treated instead. Despite of these simplifications the considered test case is representative and furthermore, the treatment of the fully coherent regime is most challenging for a discretization of the WTE. It is natural to expect that the oscillations in the Wigner function are damped as soon as scattering is included. Also due to the recent work outlined in [27,28], which questions the applicability of the inflow/outflow boundary conditions for the WTE, a further and more detailed analysis of the coherent transport regime is of interest.

The paper is organized as follows: In Section 2 we recap basic aspects of the Wigner function formalism before discussing the numerical method in Section 3 in detail. After performing an averaging of the WTE, we briefly introduce an operator splitting scheme, and thereafter, focus on the particular discretization of the pseudo-differential operator and of the advection term separately. In Section 4 we present results for the simulation of a RTD. At first, a detailed convergence study is given together with comparing the current–voltage characteristics with a NEGF computation, before presenting well-resolved phase space plots of the steady state Wigner function and results for a transient response simulation. Concluding remarks are given in Section 5.

## 2 Wigner transport equation

A possible quantum mechanical description of an open system of electrons where decoherence phenomena such as electron–phonon scattering are present, is given in terms of the von Neumann equation for the density operator $\overset{ˆ}{\rho }(t)$. Frensley [8] pointed out that it is in some cases problematic to impose appropriate boundary conditions for the density operator. By making use of the Wigner function it is possible to circumvent this disadvantage [1,6–8,29–31]. The approach is based on transforming the density matrix in position space $\rho (x^{\prime },x^{\prime \prime },t)=\langle x^{\prime }|\overset{ˆ}{\rho }(t)|x^{\prime \prime }\rangle$ into a quasi-distribution function f(p,x,t) in phase space, with $x,x^{\prime },x^{\prime \prime },p\in R$ and t>0 when considering the special case of one dimension. It is done upon employing a Fourier transformation with respect to the difference coordinate *ξ*:

(1) $$f(p,x,t)=\frac{1}{2\pi \hbar }\int_{R}\langle x+\frac{1}{2}\xi |\overset{ˆ}{\rho }(t)|x-\frac{1}{2}\xi \rangle \exp\left(-i\frac{p}{\hbar }\xi \right)d\xi ,$$

known as the Wigner–Weyl transform, which is invertible [30]. The Wigner function f(p,x,t) enables an analogous phase space description as in the case of a classical distribution function with the same moments when integrating f(p,x,t) with respect to momentum *p* and position *x*. In particular, the particle density is given as the marginal distribution

(2) $$n(x,t)=\int_{R}f(p,x,t)dp,$$

and the current density is related to the first moment by

(3) $$j(x,t)=\frac{q}{m^{⁎}}\int_{R}pf(p,x,t)dp,$$

with the electron charge q<0 and $m^{⁎}$ denoting the effective mass. However, f(p,x,t) does not share all properties of a probability distribution since it may take on negative values in some regions of the phase space and is thus termed quasi-distribution function (for an interpretation of these negative-valued regions see for instance [32]). Due to the analogy to a classical distribution function, the same boundary conditions are applicable which clearly distinguish the incoming from the outgoing part of the distribution f(p,x,t), see Section 3.4.

The dynamics of the density operator is governed by the von Neumann equation [30]

(4) $$i\hbar \partial_{t}\overset{ˆ}{\rho }=[\overset{ˆ}{H},\overset{ˆ}{\rho }],$$

where $[\overset{ˆ}{H},\overset{ˆ}{\rho }]=\overset{ˆ}{H}\overset{ˆ}{\rho }-\overset{ˆ}{\rho }\overset{ˆ}{H}$ is the commutator with the Hamiltonian. Similar to Eq. (1), it is possible to map any Hilbert space operator onto a c-number phase space function by the Weyl–Wigner correspondence [30]. For Hermitian operators such as $\overset{ˆ}{H}$, the Wigner map $\overset{ˆ}{H}\to H(p,x,t)$ results in a real-valued phase space function. For the special case of a Hamiltonian of the form $\overset{ˆ}{H}=\overset{ˆ}{E}(\overset{ˆ}{p})+q\overset{ˆ}{V}(\overset{ˆ}{x},t)$, i.e. without products of non-commuting operators, the corresponding phase space function is simply obtained by replacing the operators by their eigenvalues, H(p,x,t)=E(p)+qV(x,t) [29]. In place of the non-commutative product of operators, the so-called Moyal star product is encountered [29]

(5) $$⋆=\exp\left[i\frac{\hbar }{2}({\overset{\leftarrow }{\partial }}_{x}{\overset{\to }{\partial }}_{p}-{\overset{\leftarrow }{\partial }}_{p}{\overset{\to }{\partial }}_{x})\right],$$

where an arrow to the left (right) indicates that the derivative acts only on functions to the left-hand (right-hand) side. A product of operators is transformed as [29]

(6) $$\overset{ˆ}{H}(t)\overset{ˆ}{\rho }(t)\mapsto H(p,x,t)⋆f(p,x,t).$$

Therefore, upon applying a Wigner transformation to the von Neumann equation (4), the phase space analogue, Moyal's equation, is found [29]:

(7) $$\partial_{t}f(p,x,t)=\left\{\left\{H(p,x,t),f(p,x,t)\right\}\right\}.$$

The Moyal bracket is defined by [29,33]

(8) $$\left\{\left\{H(p,x,t),f(p,x,t)\right\}\right\}=\frac{H(p,x,t)⋆f(p,x,t)-f(p,x,t)⋆H(p,x,t)}{i\hbar }.$$

For the Hamiltonian considered in this work, Moyal's equation simplifies further. In particular we choose H(p,x,t)=E(p)+qV(x,t) with the kinetic energy term in the effective mass approximation $E(p)\approx E_{C}+\frac{p^{2}}{2m^{⁎}}$, where $E_{C}$ is the energy of the conduction band edge, and with an arbitrary electrostatic potential V(x,t). Due to this approximation, the kinetic part of the Moyal bracket reduces to

(9) $$\left\{\left\{E(p),f(p,x,t)\right\}\right\}=-\frac{p}{m^{⁎}}\partial_{x}f(p,x,t),$$

and is thus simply given by an advection term [34]. When rewriting the potential energy term in Eq. (7) in form of an equivalent integral expression, we arrive at the so-called Wigner transport equation (WTE) [1,6,35]

(10) $$\partial_{t}f(p,x,t)=-\frac{p}{m^{⁎}}\partial_{x}f(p,x,t)+\frac{q}{m^{⁎}}\left(\Theta_{\hbar }[V]f\right)(p,x,t),$$

with the pseudo-differential operator [1]

(11) $$\left(\Theta_{\hbar }[V]f\right)(p,x,t)=\frac{im^{⁎}}{2\pi \hbar }\int_{-\infty }^{\infty }\int_{-\infty }^{\infty }\frac{\left[V\left(x+\frac{\eta^{\prime }}{2},t\right)-V\left(x-\frac{\eta^{\prime }}{2},t\right)\right]}{\hbar }f\left(p^{\prime },x,t\right)\exp\left(i\frac{p-p^{\prime }}{\hbar }\eta^{\prime }\right)dp^{\prime }d\eta^{\prime }=2ℜ\left\{\;\int_{-\infty }^{\infty }\frac{im^{⁎}}{\hbar }V\left(x+\frac{\eta^{\prime }}{2},t\right)\overset{˜}{f}\left(\eta^{\prime },x,t\right)\exp\left(i\frac{p}{\hbar }\eta^{\prime }\right)d\eta^{\prime }\right\},$$

also known as the Wigner kernel, and the Fourier transformed Wigner function defined as

(12) $$\overset{˜}{f}(\eta ,x,t)=\frac{1}{2\pi \hbar }\int_{-\infty }^{\infty }f(p,x,t)\exp\left(-i\frac{p}{\hbar }\eta \right)dp.$$

When comparing the WTE to the semi-classical Boltzmann transport equation [1], it is apparent that the kinetic part is represented in the same way by an advection term but the electrostatic potential enters the WTE in a more complicated, non-local way through $\left(\Theta_{\hbar }[V]f)(p,x,t\right)$.

The pseudo-differential operator $\left(\Theta_{\hbar }[V]f)(p,x,t\right)$ acts in the Fourier transformed space as a simple multiplication of $\overset{˜}{f}(\eta ,x,t)$ and the multiplicator

(13) $${\left(\delta V\right)}_{\hbar }\left(\eta^{\prime },x,t\right)=\frac{im^{⁎}}{\hbar }\left[V\left(x+\frac{\eta^{\prime }}{2},t\right)-V\left(x-\frac{\eta^{\prime }}{2},t\right)\right],$$

which is called the symbol of the pseudo-differential operator [1].

One fundamental property of the WTE is that the continuity equation can be retrieved. A short calculation reveals that the contribution of $\left(\Theta_{\hbar }[V]f)(p,x,t\right)$ vanishes when integrating it with respect to *p*:

(14) $$\int_{-\infty }^{\infty }\left(\Theta_{\hbar }[V]f\right)(p,x,t)dp=0.$$

When making use of the relations of f(p,x,t) to n(x,t) and j(x,t), Eqs. (2) and (3), respectively, and integrating the whole WTE (10) with respect to *p*, the continuity equation is readily obtained:

(15) $$q\partial_{t}n(x,t)+\partial_{x}j(x,t)=0.$$

## 3 Numerical method

### 3.1 Averaging the Wigner transport equation

In the following we average the Wigner transport equation over grid cells defined by

(16) $$C_{m,j}=C_{m}^{\left(p\right)}\times C_{j}^{\left(x\right)},$$

with

(17) $$C_{m}^{\left(p\right)}=(p_{m-1/2},p_{m+1/2}),\;m=1,\ldots ,N_{p},$$

$$C_{j}^{\left(x\right)}=(x_{j-1/2},x_{j+1/2}),\;j=1,\ldots ,N_{x}.$$

In order to construct a conservative method, we also need the half-bounded intervals

(18) $$C_{0,j}=C_{0}^{\left(p\right)}\times C_{j}^{\left(x\right)},\;j=1,\ldots ,N_{x},$$

$$C_{N_{p}+1,j}=C_{N_{p}+1}^{\left(p\right)}\times C_{j}^{\left(x\right)},\;j=1,\ldots ,N_{x},$$

with

(19) $$C_{0}^{\left(p\right)}=(-\infty ,p_{1/2}),$$

$$C_{N_{p}+1}^{\left(p\right)}=(p_{N_{p}+1/2},\infty ).$$

The grid for the *x* variable is chosen to be equidistant but all of the following derivations can be directly adopted to a non-equidistant *x* grid. No constraints are assumed for the grid spacing of the *p* variable, which enables a highly flexible and adaptable grid for different physical situations. In particular, the cell boundaries for the *x* and *p* grids are defined by

(20) $$x_{j-1/2}=x_{1/2}+(j-1)\Delta x,\;x_{1/2}\in R,\;\Delta x\in R,\;j=1,\ldots ,N_{x}+1,$$

$$p_{m-1/2}=p_{1/2}+\sum_{l=1}^{m-1}\Delta p_{l},\;p_{1/2}\in R,\;\Delta p_{l}\in R,\;m=2,\ldots ,N_{p}+1.$$

The central points of the grid cells are labeled by integer indices and given by

(21) $$x_{j}=\frac{x_{j-1/2}+x_{j+1/2}}{2},\;j=1,\ldots ,N_{x},$$

$$p_{m}=\frac{p_{m-1/2}+p_{m+1/2}}{2},\;m=1,\ldots ,N_{p}.$$

The particular approximations used for the Wigner function will be discussed later in Sections 3.3 and 3.4. At this point we only demand that f(p,x,t)=0 for $\left(p,x)\notin (p_{1/2},p_{N_{p}+1/2})\times (x_{1/2},x_{N_{x}+1/2}\right)$. This restriction to a compact support of f(p,x,t) may introduce finite size errors in actual computations but can always be controlled by increasing the domain size, as demonstrated in Section 4.2. The cell averages of the Wigner function over the grid cells $C_{m,j}$ are given by

(22) $$F_{m,j}(t)=\frac{1}{\Delta p_{m}\Delta x}\iint_{C_{m,j}}f(p,x,t)dpdx,\;m=1,\ldots ,N_{p},\;j=1,\ldots ,N_{x}.$$

To arrive at a conservative scheme, we now average the whole Wigner transport equation (10) over grid cells. For the interior grid cells $C_{m,j}$, with interior referring to the *p* variable, we arrive at

(23) $$\partial_{t}F_{m,j}(t)=\frac{1}{\Delta p_{m}\Delta x}\iint_{C_{m,j}}\left[-\frac{p}{m^{⁎}}\;\partial_{x}f(p,x,t)+\frac{q}{m^{⁎}}\left(\Theta_{\hbar }[V]f\right)(p,x,t)\right]dpdx,$$

with $m=2,\ldots ,N_{p}-1,\;\;j=1,\ldots ,N_{x}$, where the left-sided term was identified as the time derivative of the cell average. To obtain the governing equations for $\partial_{t}F_{1,j}(t)$ ($\partial_{t}F_{N_{p},j}(t)$), we consider the corresponding finite intervals $C_{1,j}$ ($C_{N_{p},j}$) together with the semi-infinite *p* intervals $C_{0,j}$ ($C_{N_{p}+1,j}$):

(24) $$\iint_{C_{1,j}⋃C_{0,j}}\left[\partial_{t}f(p,x,t)+\frac{p}{m^{⁎}}\;\partial_{x}f(p,x,t)\right]dpdx=\iint_{C_{1,j}⋃C_{0,j}}\frac{q}{m^{⁎}}\left(\Theta_{\hbar }[V]f\right)(p,x,t)dpdx.$$

Since f(p,x,t) is assumed to vanish outside of $\left(p_{1/2},p_{N_{p}+1/2})\times (x_{1/2},x_{N_{x}+1/2}\right)$, the left-hand side of Eq. (24) reduces to

(25) $$\iint_{C_{1,j}}\left[\partial_{t}f(p,x,t)+\frac{p}{m^{⁎}}\;\partial_{x}f(p,x,t)\right]dpdx=\Delta p_{1}\Delta x\partial_{t}F_{1,j}(t)+\iint_{C_{1,j}}\frac{p}{m^{⁎}}\;\partial_{x}f(p,x,t)dpdx,$$

whereas we need to consider for the right-hand side the integration over the full semi-infinite *p* interval. On the whole, this results in the following expression for the boundary terms m=1:

(26) $$\partial_{t}F_{1,j}(t)=\frac{1}{\Delta p_{1}\Delta x}\int_{x_{j-1/2}}^{x_{j+1/2}}\;\left[\;\int_{p_{1/2}}^{p_{3/2}}\left(-\frac{p}{m^{⁎}}\right)\partial_{x}f(p,x,t)dp+\int_{-\infty }^{p_{3/2}}\frac{q}{m^{⁎}}\left(\Theta_{\hbar }[V]f\right)(p,x,t)dp\right]dx,$$

and for the case $m=N_{p}$ we obtain:

(27) $$\partial_{t}F_{N_{p},j}(t)=\frac{1}{\Delta p_{N_{p}}\Delta x}\int_{x_{j-1/2}}^{x_{j+1/2}}\;\left[\;\int_{p_{N_{p}-1/2}}^{p_{N_{p}+1/2}}\left(-\frac{p}{m^{⁎}}\right)\partial_{x}f(p,x,t)dp+\int_{p_{N_{p}-1/2}}^{\infty }\frac{q}{m^{⁎}}\left(\Theta_{\hbar }[V]f\right)(p,x,t)dp\right]dx.$$

The equations stated so far suffice to show that the numerical scheme developed in this work preserves the particle density, independent of the particular discretization of the momentum grid. From the knowledge of the cell averages, the averaged particle density in the interval $\left(x_{j-1/2},x_{j+1/2}\right)$ is calculated by

(28) $$\overset{¯}{n}(x_{j},t)=\sum_{m=1}^{N_{p}}F_{m,j}(t)\Delta p_{m}.$$

When inserting the expressions for the time derivatives of the cell averages, Eqs. (23), (26) and (27), into the time derivative of Eq. (28), we arrive at

(29) $$\partial_{t}\overset{¯}{n}(x_{j},t)=\frac{1}{\Delta x}\int_{x_{j-1/2}}^{x_{j+1/2}}\int_{-\infty }^{\infty }\frac{q}{m^{⁎}}\left(\Theta_{\hbar }[V]f\right)(p,x,t)dpdx-\sum_{m=1}^{N_{p}}\frac{1}{\Delta x}\int_{C_{m}^{\left(p\right)}}\frac{p}{m^{⁎}}\left[f(p,x_{j+1/2},t)-f(p,x_{j-1/2},t)\right]dp=-\frac{1}{\Delta x}\int_{p_{1/2}}^{p_{N_{p}+1/2}}\frac{p}{m^{⁎}}\left[f(p,x_{j+1/2},t)-f(p,x_{j-1/2},t)\right]dp,$$

where the property Eq. (14) was made use of. As can be seen, the action of the pseudo-differential operator does not influence the particle density $\overset{¯}{n}(x_{j},t)$ and the expression in the last line corresponds to the net flux into the interval $\left(x_{j-1/2},x_{j+1/2}\right)$. This tells us that the averaging procedure outlined here preserves the particle density and is consistent with the continuity equation.

### 3.2 Operator splitting

In the following two sections the numerical scheme is formulated in terms of the cell averages $F_{m,j}(t)$. This is done by choosing for f(p,x,t) a certain approximation for each operator, which is uniquely determined by the set of cell averages. When introducing the vector notation

(30) $$F(t)=\left(F_{1,1}(t),\ldots ,F_{1,N_{x}}(t),\ldots ,F_{N_{p},1}(t),\ldots ,F_{N_{p},N_{x}}(t)\right),$$

the averaged WTE, Eqs. (23), (26) and (27), is written in abstract notation as

(31) $$\partial_{t}F(t)=(L_{A}+L_{V})F(t),$$

where the operators $L_{A}$ and $L_{V}$ correspond to the ones resulting from the advection term and from the pseudo-differential operator, respectively. For the case that $L_{A}$ and $L_{V}$ are not explicitly time-dependent, the formal solution is given by

(32) $$F(t)=\left(\prod_{i=1}^{(t-t_{0})/\Delta t}\exp\left[(L_{A}+L_{V})\Delta t\right]\right)F(t_{0}).$$

By employing a so-called Strang splitting [36,37], the time evolution due to the distinct operators can be treated separately, one after the other:

(33) $$F\left(t^{\prime }+\Delta t\right)=\exp\left(L_{A}\frac{\Delta t}{2}\right)\exp[L_{V}\Delta t]\exp\left(L_{A}\frac{\Delta t}{2}\right)F\left(t^{\prime }\right)+O\left(\Delta t^{3}\right)F\left(t^{\prime }\right).$$

The error encountered in each time step Δ*t* is proportional to the commutator $\left[L_{A},L_{V}\right]$ and to $\Delta t^{3}$, such that the overall accuracy is of second order in Δ*t*. The advantage of employing an operator splitting is that appropriate time stepping methods can be used for the individual operators in the three sub-time steps in Eq. (33).

### 3.3 Discretization of the pseudo-differential operator

In the following we focus on the sub-problem of discretizing the pseudo-differential operator, starting from Eqs. (23), (26) and (27) but leaving out the advection term. For each interior grid cell, the integration with respect to *p* in Eq. (23) is taken over a bounded interval, thus conceptually not difficult and results in

(34) $$\partial_{t}F_{m,j}(t)=\frac{1}{\Delta p_{m}\Delta x}ℜ\left\{\frac{2q}{\hbar }\int_{x_{j-1/2}}^{x_{j+1/2}}\int_{-\infty }^{\infty }V\left(x+\frac{\eta^{\prime }}{2},t\right)\overset{˜}{f}\left(\eta^{\prime },x,t\right)\frac{\hbar }{\eta^{\prime }}\left[\exp\left(i\frac{p_{m+1/2}}{\hbar }\eta^{\prime }\right)-\exp\left(i\frac{p_{m-1/2}}{\hbar }\eta^{\prime }\right)\right]d\eta^{\prime }dx\right\},\;m=2,\ldots ,N_{p}-1,\;j=1,\ldots ,N_{x},$$

where the definition of the pseudo-differential operator, Eq. (11), was used. To perform the integration over the semi-infinite *p* intervals in Eqs. (26) and (27) we recall the Fourier transform of the Heaviside step function, given by [38]

(35) $$\int_{-\infty }^{\infty }H(t)\exp(-i\omega t)dt=\mathrm{PV}\frac{1}{i\omega }+\pi \delta (\omega ),$$

with PV denoting the principal value, so that we obtain for the case of the lower boundary

(36) $$\partial_{t}F_{1,j}(t)=\frac{1}{\Delta p_{1}\Delta x}ℜ\left\{\frac{2iq}{\hbar }\int_{x_{j-1/2}}^{x_{j+1/2}}\int_{-\infty }^{\infty }V\left(x+\frac{\eta^{\prime }}{2},t\right)\overset{˜}{f}\left(\eta^{\prime },x,t\right)\left[-i\frac{\hbar }{\eta^{\prime }}\exp\left(i\frac{p_{3/2}}{\hbar }\eta^{\prime }\right)+\hbar \pi \delta \left(\eta^{\prime }\right)\right]d\eta^{\prime }dx\right\}=\frac{1}{\Delta p_{1}\Delta x}ℜ\left\{\frac{2q}{\hbar }\int_{x_{j-1/2}}^{x_{j+1/2}}\int_{-\infty }^{\infty }V\left(x+\frac{\eta^{\prime }}{2},t\right)\overset{˜}{f}\left(\eta^{\prime },x,t\right)\frac{\hbar }{\eta^{\prime }}\exp\left(i\frac{p_{3/2}}{\hbar }\eta^{\prime }\right)d\eta^{\prime }dx\right\},\;j=1,\ldots ,N_{x},$$

since $\overset{˜}{f}(\eta^{\prime }\;=\;0,x,t)\in R$. An analogous result is found for the upper boundary $m=N_{p}$, in which $p_{3/2}$ is replaced by $p_{N_{p}-1/2}$ and an overall minus sign occurs.

After this general considerations, we restrict ourselves to Wigner functions which are representable as piecewise polynomials with respect to *p* and *x*. In particular we choose

(37) $$f(p,x,t)=\sum_{m=1}^{N_{p}}\sum_{j=1}^{N_{x}}P_{m,j}^{\gamma }(p,x,t).$$

In principle one is not limited in the order *γ* of the polynomials, but in this work we consider at most a piecewise constant approximation with respect to *x* and first-order polynomials with respect to *p*, given by

(38) $$P_{m,j}^{1}(p,x,t):=\left\{ \begin{array}{cc} F_{m,j}(t)+\sigma_{m,j}(t)(p-p_{m}) & \text{if }(p,x)\in C_{m,j},\;m\neq \{0,N_{p}+1\},\\ 0 & \text{elsewhere}. \end{array}\right.$$

The slopes $\sigma_{m,j}(t)$ are determined from the cell averages $F_{m,j}(t)$ by central finite differences with respect to *p* at the interior grid points and by one-sided finite differences at the boundaries $m=1,N_{p}$. Since a direct numerical evaluation of the oscillatory integrals involved in $\left(\Theta_{\hbar }[V]f)(p,x,t\right)$ is problematic, we also choose for V(x,t) a certain basis representation. To be specific, piecewise linear polynomials are used as well:

(39) $$V(x,t)=\sum_{k=0}^{N_{V}}p_{k}^{V}(x,t),$$

with

(40) $$p_{k}^{V}(x,t):=\left\{ \begin{array}{cc} V_{k}(t)+\frac{V_{k+1}(t)-V_{k}(t)}{x_{k+1}^{V}-x_{k}^{V}}(x-x_{k}^{V}) & \text{if }x_{k}^{V}\leq x<x_{k+1}^{V},\\ 0 & \text{elsewhere}. \end{array}\right.$$

**Fig. 1:**
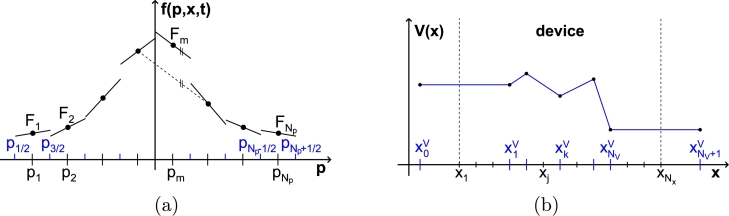
Schematic drawings of the piecewise linear approximations chosen for the Wigner function in (a) and for the potential in (b).

Here, a continuous form of V(x,t) is chosen but it should be mentioned that this is not mandatory. The grid points for the potential are selected to be a subset of the grid points for the Wigner function,

(41) $$\left\{x_{k}^{V}|k=1,\ldots ,N_{V}\right\}\subseteq \{x_{j}|j=1,\ldots ,N_{x}\},$$

with $N_{V}\leq N_{x}$. Furthermore, two additional grid points outside the *x* interval for f(p,x,t) are introduced, $x_{0}^{V}$ and $x_{N_{V}+1}^{V}$ with $V_{0}(t)=V_{1}(t)$ and $V_{N_{V}+1}(t)=V_{N_{V}}(t)$, which we will let go to ±∞ in the final equations to model the situation of semi-infinite leads under bias. Schematic drawings of the approximate forms chosen for f(p,x,t) and V(x,t) are depicted in Fig. 1.

Inserting the particular approximations for f(p,x,t) and V(x,t) into Eqs. (34) and (36) results in

(42) $$\partial_{t}F_{m,j}(t)=\frac{1}{\Delta p_{m}\Delta x}\frac{2q}{\hbar }\sum_{k=0}^{N_{V}}\;\int_{x_{j-1/2}}^{x_{j+1/2}}\int_{2(x_{k}^{V}-x)}^{2(x_{k+1}^{V}-x)}\left[V_{k}(t)+\frac{V_{k+1}(t)-V_{k}(t)}{x_{k+1}^{V}-x_{k}^{V}}\left(x+\frac{\eta^{\prime }}{2}-x_{k}^{V}\right)\right]K_{m,j}\left(\eta^{\prime },t\right)d\eta^{\prime }dx,$$

with

(43) $$K_{m,j}\left(\eta^{\prime },t\right):=\left\{ \begin{array}{cc} ℜ\{\overset{˜}{f}(\eta^{\prime },x_{j},t)\frac{\hbar }{\eta^{\prime }}[\exp(i\frac{p_{m+1/2}}{\hbar }\eta^{\prime })-\exp(i\frac{p_{m-1/2}}{\hbar }\eta^{\prime })]\} & \text{for }m=2,\ldots ,N_{p}-1,\\ ℜ\{\overset{˜}{f}(\eta^{\prime },x_{j},t)\frac{\hbar }{\eta^{\prime }}\exp(i\frac{p_{3/2}}{\hbar }\eta^{\prime })\} & \text{for }m=1,\\ ℜ\{-\overset{˜}{f}(\eta^{\prime },x_{j},t)\frac{\hbar }{\eta^{\prime }}\exp(i\frac{p_{N_{p}-1/2}}{\hbar }\eta^{\prime })\} & \text{for }m=N_{p}, \end{array}\right.$$

and the Fourier transform $\overset{˜}{f}(\eta^{\prime },x_{j},t)$ on the interval $\left(x_{j-1/2},x_{j+1/2}\right)$ given by

(44) $$\overset{˜}{f}\left(\eta^{\prime },x_{j},t\right)=\frac{1}{2\pi \hbar }\sum_{i=1}^{N_{p}}\int_{p_{i-1/2}}^{p_{i+1/2}}\left[F_{i,j}(t)+\sigma_{i,j}(t)\left(p^{\prime }-p_{i}\right)\right]\exp\left(-i\frac{p^{\prime }}{\hbar }\eta^{\prime }\right)dp^{\prime }.$$

The integrations in Eq. (42) are carried out analytically, as far as possible at least, since sine and cosine integrals appear. In practice, standard routines are used to evaluate the trigonometric integrals numerically. After lengthy but conceptually not difficult calculations, for details see Appendix A, one arrives at the following set of equations:

(45) $$\partial_{t}F_{m,j}(t)=\sum_{i=1}^{N_{p}}D_{m,i,j}^{F}(t)F_{i,j}(t)+\sum_{i=1}^{N_{p}}D_{m,i,j}^{\sigma }(t)\sigma_{i,j}(t),\;m=1,\ldots ,N_{p},\;j=1,\ldots ,N_{x},$$

with the matrix elements given by

(46) $$D_{m,i,j}^{F}=\frac{q}{\pi \Delta p_{m}\Delta x}\left[I_{m+1/2,i+1/2,j}^{F}-I_{m+1/2,i-1/2,j}^{F}-\left(I_{m-1/2,i+1/2,j}^{F}-I_{m-1/2,i-1/2,j}^{F}\right)\right],\;m=2,\ldots ,N_{p}-1,$$

$$D_{1,i,j}^{F}=\frac{q}{\pi \Delta p_{1}\Delta x}\left(I_{3/2,i+1/2,j}^{F}-I_{3/2,i-1/2,j}^{F}\right),$$

$$D_{N_{p},i,j}^{F}=-\frac{q}{\pi \Delta p_{N_{p}}\Delta x}\left(I_{N_{p}-1/2,i+1/2,j}^{F}-I_{N_{p}-1/2,i-1/2,j}^{F}\right),$$

and

(47) $$D_{m,i,j}^{\sigma }=\frac{q}{\pi \Delta p_{m}\Delta x}\left[I_{m+1/2,i^{+},j}^{\sigma }-I_{m+1/2,i^{-},j}^{\sigma }-\left(I_{m-1/2,i^{+},j}^{\sigma }-I_{m-1/2,i^{-},j}^{\sigma }\right)\right],\;m=2,\ldots ,N_{p}-1,$$

$$D_{1,i,j}^{\sigma }=\frac{q}{\pi \Delta p_{1}\Delta x}\left(I_{3/2,i^{+},j}^{\sigma }-I_{3/2,i^{-},j}^{\sigma }\right),$$

$$D_{N_{p},i,j}^{\sigma }=-\frac{q}{\pi \Delta p_{N_{p}}\Delta x}\left(I_{N_{p}-1/2,i^{+},j}^{\sigma }-I_{N_{p}-1/2,i^{-},j}^{\sigma }\right).$$

The abbreviations stand for

(48) $$I_{m+1/2,i+1/2,j}^{F}=\frac{1}{k_{m,i}}\sum_{k=1}^{N_{V}-1}\frac{V_{k+1}(t)-V_{k}(t)}{x_{k+1}^{V}-x_{k}^{V}}\left[T^{F,v^{1}}(u){|}_{C_{m,i,j}^{x_{k+1}^{V}}}-T^{F,v^{1}}(u){|}_{C_{m,i,j}^{x_{k}^{V}}}\right],$$

$$I_{m+1/2,i^{+},j}^{\sigma }=\sum_{k=1}^{N_{V}-1}\frac{V_{k+1}(t)-V_{k}(t)}{x_{k+1}^{V}-x_{k}^{V}}\left\{\hbar \left[T^{\sigma ,v^{1}}(u){|}_{C_{m,i,j}^{x_{k+1}^{V}}}-T^{\sigma ,v^{1}}(u){|}_{C_{m,i,j}^{x_{k}^{V}}}\right]+\left(\frac{p_{m+1/2}+p_{i+1/2}}{2}-p_{i}\right)\frac{1}{k_{m,i}}\left[T^{F,v^{1}}(u){|}_{C_{m,i,j}^{x_{k+1}^{V}}}-T^{F,v^{1}}(u){|}_{C_{m,i,j}^{x_{k}^{V}}}\right]\right\},$$

whereby the expressions

(49) $$T^{F,v^{1}}(u)=\frac{1}{8}\left[-u^{2}\mathrm{Ci}\left(|u|\right)+2u\mathrm{Si}(u)+u\sin(u)+\cos(u)\right],$$

$$T^{\sigma ,v^{1}}(u)=\frac{1}{8}\left[\mathrm{Ci}\left(|u|\right)+u\mathrm{Si}(u)+\cos(u)\right]$$

contain the sine and cosine integrals, see Appendix A. The intervals are given by

(50) $$C_{m,i,j}^{x_{k}^{V}}=\left(2k_{m,i}\left(x_{k}^{V}-x_{j-1/2}\right),2k_{m,i}\left(x_{k}^{V}-x_{j+1/2}\right)\right),$$

with

(51) $$k_{m,i}=\frac{p_{m+1/2}-p_{i+1/2}}{\hbar }.$$

The corresponding expression for $I_{m+1/2,i^{-},j}^{\sigma }$ is readily obtained from Eq. (48) by the replacements $p_{i+1/2}\to p_{i-1/2}$, $k_{m,i}\to k_{m,i-1}$ and leaving $p_{i}$ the same. For an actual implementation it is important to know the limits

(52) $$\lim_{k\to 0}\frac{1}{k}T^{F,v^{1}}(u){|}_{kd_{1}}^{kd_{2}}=0,$$

$$\lim_{k\to 0}T^{\sigma ,v^{1}}(u){|}_{kd_{1}}^{kd_{2}}=\frac{1}{8}\left[\ln|d_{2}|-\ln|d_{1}|\right],$$

with *k* representing some wavenumber, and $d_{1}$ and $d_{2}$ labeling two distances. They are needed for the diagonal terms where $k_{m,i}=0$ occurs, for more details see Appendix A.

After having stated the governing equations, we now focus on the time stepping. The set of Eqs. (45) together with the chosen linear dependence of the slopes $\sigma_{i,j}(t)$ on the cell averages $F_{i,j}(t)$ may be rewritten as

(53) $$\partial_{t}F_{m,j}(t)=\sum_{i=1}^{N_{p}}D_{m,i,j}F_{i,j}(t),\;m=1,\ldots ,N_{p},\;j=1,\ldots ,N_{x}.$$

The formal solution to advance from time step $t_{n}$ to $t_{n+1}=t_{n}+\Delta t$ is given by the system

(54) $$F_{m,j}^{n+1}=\sum_{i=1}^{N_{p}}\exp{\left(\Delta tD\right)}_{m,i,j}F_{i,j}^{n},\;m=1,\ldots ,N_{p},$$

for each spatial index $j=1,\ldots ,N_{x}$. In order to evaluate the matrix exponential exp⁡(ΔtD) numerically, we make use of the scaling and squaring method [39]. For steady state or transient response simulations, as considered in this work, the extra computational cost for evaluating the matrix exponential is negligible. Fully time dependent problems on the other hand require different approaches, such as a Runge–Kutta scheme for instance.

### 3.4 Discretization of the advection term

In the present section we focus on the advection term and consider the following sub-problem of the averaged WTE, Eqs. (23), (26) and (27):

(55) $$\partial_{t}F_{m,j}(t)=\frac{1}{\Delta p_{m}\Delta x}\iint_{C_{m,j}}\left(-\frac{p}{m^{⁎}}\right)\partial_{x}f(p,x,t)dpdx,$$

with $m=1,\ldots ,N_{p}$, $j=1,\ldots ,N_{x}$. This can be rewritten as

(56) $$\partial_{t}F_{m,j}(t)=-\frac{1}{\Delta x}\left[h_{m,j+1/2}(t)-h_{m,j-1/2}(t)\right],$$

with the flux at the two spatial boundaries of grid cell $C_{m,j}$ given by

(57) $$h_{m,j\pm 1/2}(t)=\frac{1}{\Delta p_{m}}\int_{p_{m-1/2}}^{p_{m+1/2}}\frac{p}{m^{⁎}}f(p,x_{j\pm 1/2},t)dp.$$

The advection term is acting via $\partial_{x}$ only on the *x* coordinate of f(p,x,t), so that it is convenient to approximate f(p,x,t) in this term as a piecewise constant function with respect to *p*. With respect to *x* some higher-order approximation will be chosen. The assumption that f(p,x,t) is constant on the interval $\left(p_{m-1/2},p_{m+1/2}\right)$ greatly simplifies Eq. (57) and we arrive at

(58) $$h_{m,j\pm 1/2}(t)=\frac{p_{m}}{m^{⁎}}f(p_{m},x_{j\pm 1/2},t).$$

Eqs. (56) decouple now with respect to *m* and the problem reduces to solving a set of $N_{p}$ one-dimensional advection equations. To express the flux $h_{m,j+1/2}(t)$ in terms of the cell averages $F_{m,j}(t)$, one has to rely on approximate schemes. High-order methods without slope or flux limiters [34,37] were found to be problematic, in the coherent transport regime at least, due to the creation of spurious oscillations. We obtained good results with a MC limiter (monotonized central-difference) scheme [40], but found the convergence rate of the method to be rather low, especially in regions with strong variations of V(x,t) and thus of f(p,x,t) as well. Since the problem of strong spatial variations of the distribution function is common in device simulations due to steep doping profiles, see e.g. [2], well developed schemes exist that can cope with such cases, known as weighted essentially non-oscillatory (WENO) methods. In this procedure, the numerical fluxes are obtained as the convex sum of a certain number of approximations on different stencils, where the corresponding weights are adjusted to the smoothness of the distribution function. The first such methods were developed in [24]. In this work we will focus on the WENO5 scheme presented in [2] or [25]. The method and especially its stability properties were extensively analyzed in [41].

In the WENO5 scheme, three different stencils are used to approximate the flux such that $h_{m,j+1/2}(t_{n})$ (Eq. (58)) at time $t_{n}$ is given by

(59) $$h_{m,j+1/2}^{n}=\omega_{1}^{n}h_{m,j+1/2}^{n,(1)}+\omega_{2}^{n}h_{m,j+1/2}^{n,(2)}+\omega_{3}^{n}h_{m,j+1/2}^{n,(3)}.$$

For the case $p_{m}>0$ the three single fluxes are determined by

(60) $$h_{m,j+1/2}^{n,(1)}=\frac{p_{m}}{m^{⁎}}\left(\frac{1}{3}F_{m,j-2}^{n}-\frac{7}{6}F_{m,j-1}^{n}+\frac{11}{6}F_{m,j}^{n}\right),$$

$$h_{m,j+1/2}^{n,(2)}=\frac{p_{m}}{m^{⁎}}\left(-\frac{1}{6}F_{m,j-1}^{n}+\frac{5}{6}F_{m,j}^{n}+\frac{1}{3}F_{m,j+1}^{n}\right),$$

$$h_{m,j+1/2}^{n,(3)}=\frac{p_{m}}{m^{⁎}}\left(\frac{1}{3}F_{m,j}^{n}+\frac{5}{6}F_{m,j+1}^{n}-\frac{1}{6}F_{m,j+2}^{n}\right).$$

The single weights $\omega_{i}^{n}$ are normalized by the equation

(61) $$\omega_{i}^{n}=\frac{{\overset{˜}{\omega }}_{i}^{n}}{\sum_{l=1}^{3}{\overset{˜}{\omega }}_{l}^{n}},\;i=1,2,3,$$

with

(62) $${\overset{˜}{\omega }}_{i}^{n}=\frac{\gamma_{i}}{{\left(\varepsilon +\beta_{i}^{n}\right)}^{2}},\;i=1,2,3,$$

where the values of $\gamma_{i}$ are fixed by $\gamma_{1}=\frac{1}{10}$, $\gamma_{2}=\frac{3}{5}$ and $\gamma_{3}=\frac{3}{10}$. The small quantity $\varepsilon \approx 10^{-6}$ prevents the denominator to vanish. The smoothness indicators $\beta_{i}^{n}$ are determined by the following equations

(63) $$\beta_{1}^{n}={\left(\frac{p_{m}}{m^{⁎}}\right)}^{2}\left[\frac{13}{12}{\left(F_{m,j-2}^{n}-2F_{m,j-1}^{n}+F_{m,j}^{n}\right)}^{2}+\frac{1}{4}{\left(F_{m,j-2}^{n}-4F_{m,j-1}^{n}+3F_{m,j}^{n}\right)}^{2}\right],$$

$$\beta_{2}^{n}={\left(\frac{p_{m}}{m^{⁎}}\right)}^{2}\left[\frac{13}{12}{\left(F_{m,j-1}^{n}-2F_{m,j}^{n}+F_{m,j+1}^{n}\right)}^{2}+\frac{1}{4}{\left(F_{m,j-1}^{n}-F_{m,j+1}^{n}\right)}^{2}\right],$$

$$\beta_{3}^{n}={\left(\frac{p_{m}}{m^{⁎}}\right)}^{2}\left[\frac{13}{12}{\left(F_{m,j}^{n}-2F_{m,j+1}^{n}+F_{m,j+2}^{n}\right)}^{2}+\frac{1}{4}{\left(3F_{m,j}^{n}-4F_{m,j+1}^{n}+F_{m,j+2}^{n}\right)}^{2}\right].$$

The fluxes and smoothness indicators for $p_{m}<0$ are constructed in an analogous way.

In order to treat the boundaries appropriately, ghost cells [34] are introduced which specify values for $F_{m,j}^{n}$ at the positions j=−2,−1,0 and $j=N_{x}+1,N_{x}+2,N_{x}+3$. Boundary conditions for ohmic contacts are applied as described in [2]. For the case of the left-sided contact this results in

(64) $$F_{m,j}^{n}=\left\{ \begin{array}{cc} \frac{1}{\Delta p_{m}}\int_{p_{m-1/2}}^{p_{m+1/2}}f_{l}(p)dp & \text{if }p_{m}>0,\\ F_{m,1}^{n} & \text{if }p_{m}<0, \end{array}\right.$$

for j=0,−1,−2 and with $f_{l}(p)$ labeling the one-dimensional Fermi–Dirac distribution of the left contact, see [8,42,43]. The values for the ghost cells on the right-sided reservoir are determined in an analogous way.

To perform the time step, a third-order Runge Kutta method termed SSP(3,3) is employed, motivated by the work of Wang [41]. In this method three sub-steps are needed to advance one complete time step and the abbreviation SSP stands for the strong stability preserving property. When written in terms of the fluxes, the three sub-steps of the SSP(3,3) algorithm consist of

(65) $$F_{m,j}^{n^{\prime }}=F_{m,j}^{n}-\frac{\Delta t}{\Delta x}\left[h_{m,j+1/2}^{n}-h_{m,j-1/2}^{n}\right],$$

$$F_{m,j}^{n^{\prime \prime }}=\frac{1}{4}\left(3F_{m,j}^{n}+F_{m,j}^{n^{\prime }}\right)-\frac{1}{4}\frac{\Delta t}{\Delta x}\left[h_{m,j+1/2}^{n^{\prime }}-h_{m,j-1/2}^{n^{\prime }}\right],$$

$$F_{m,j}^{n+1}=\frac{1}{3}\left(F_{m,j}^{n}+2F_{m,j}^{n^{\prime \prime }}\right)-\frac{2}{3}\frac{\Delta t}{\Delta x}\left[h_{m,j+1/2}^{n^{\prime \prime }}-h_{m,j-1/2}^{n^{\prime \prime }}\right],$$

where the individual fluxes $h_{m,j+1/2}^{n}$, $h_{m,j+1/2}^{n^{\prime }}$ and $h_{m,j+1/2}^{n^{\prime \prime }}$ are determined by the WENO5 scheme, Eqs. (59)–(63), out of the set of cell averages $F_{m,j}^{n}$, $F_{m,j}^{n^{\prime }}$ and $F_{m,j}^{n^{\prime \prime }}$, respectively.

## 4 Results for the simulation of a resonant tunneling diode

### 4.1 Device under consideration and methodology

**Fig. 2:**
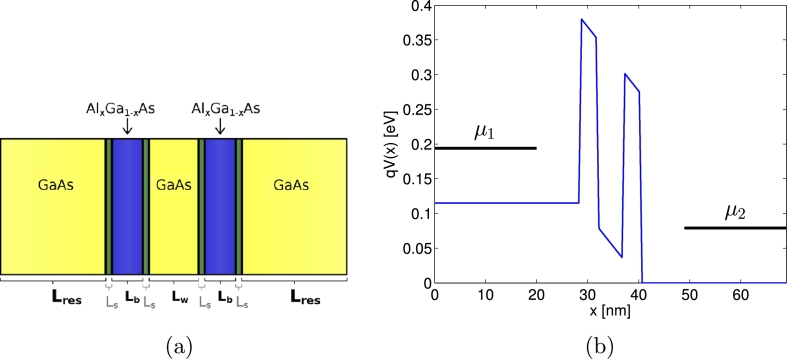
Illustration of the considered RTD consisting of an AlGaAs–GaAs-heterostructure in (a) and the corresponding potential *qV*(*x*) in (b). The plot of *qV*(*x*) depicts the case of a rather short reservoir length of $L_{\mathrm{res}}=50\;a$ and a bias voltage of $V_{\mathrm{DS}}=0.115\;\text{V}$. The chemical potentials $\mu_{1}=0.194\;\text{eV}$ and $\mu_{2}=0.079\;\text{eV}$ of each reservoir are indicated.

As a particular test case, we consider a resonant tunneling diode (RTD) consisting of an AlGaAs–GaAs-heterostructure, as depicted schematically in Fig. 2(a). The AlGaAs–GaAs-RTD is a standard problem in the field of device simulations to benchmark numerical methods for quantum transport calculations, see for instance [8,23,44,45]. Here, we restrict ourselves to the simplest case of a homogeneous doping profile, without aiming for a self-consistent solution by coupling the WTE to Poisson's equation. We assume that the bias voltage $V_{\mathrm{DS}}$ causes a linear potential drop in the region of the double barrier and that qV(x) stays constant within the reservoirs, see Fig. 2(b). For the specific composition Al_0.3_Ga_0.7_As, the energy band offset has a magnitude of 0.27 eV [23]. The length of the reservoirs included in the *x* domain for the Wigner function is labeled by $L_{\mathrm{res}}$ and varied in different simulations. Furthermore, expressed in terms of the lattice constant a=0.565 nm of GaAs, we choose for the barriers a width of $L_{b}=5\;a$ and a slope of length $L_{s}=1\;a$, as well as $L_{w}=8\;a$ for the size of the well. A spatial dependence of the effective mass is neglected and the value $m^{⁎}=0.067\;m_{e}$ for GaAs is used on the whole *x* domain. The electron distributions and the chemical potentials $\mu_{1}$, $\mu_{2}$ inside the contacts are fixed by choosing a donor density of $N_{D}=2\times 10^{18}\;{\text{cm}}^{-3}$ and a temperature of T=300 K [8,44]. From the knowledge of the chemical potentials and temperatures for each reservoir, the cell averages of the ghost cells are set according to Eq. (64).

The validity of semi-classical boundary conditions for the WTE as introduced in [8] is a topic under vivid debate, especially after recent works which address the non-uniqueness and the symmetry properties of the Wigner function [27,28,46]. The numerical test cases presented therein are for symmetric potentials for which we cannot provide reliable, i.e. well-resolved, results due to the presence of singular terms in the steady state Wigner functions, see Section 4.3. Other recent studies demonstrate the convergence of the WTE calculations upon increasing the size of the simulation domain [44] as well as possible improvements by adapting the boundary distribution to the physical state of the active device region [47]. Despite their approximate nature we employ inflow/outflow boundary conditions here as well and demonstrate that accurate and physically valid results can be achieved for sufficiently large values of $L_{\mathrm{res}}$. Due to the problematics with singular terms we present simulations only for non-zero bias voltages $V_{\mathrm{DS}}\neq 0\;\text{V}$.

In order to evaluate the accuracy of the numerical method developed in this work, we compare the steady state j(V) curves and particle densities n(x) against a non-equilibrium Green's functions (NEGF) calculation. Details on the NEGF method may be found for instance in [3,5,42,45,48–50]. The NEGF simulations were performed with a grid spacing Δx=a/64, for which we know that the calculated quantities are well converged.

For the WTE simulations, we choose an equidistant grid for the *x* variable with different values for the spacing Δ*x* and the reservoir length $L_{\mathrm{res}}$. For the *p* grid we make extensive use of the possibility to apply a non-equidistant spacing, in order to resolve all the oscillation patterns of f(p,x,t) well enough. We specify a certain $p_{\max}$ which determines the *p* domain by $\left(-p_{\max},p_{\max}\right)$, as well as a maximum spacing $\Delta p_{\max}$ for the outermost region of the *p* grid. All the other $\Delta p_{i}$ of the interior subdivisions are then expressed as a fixed fraction of $\Delta p_{\max}$. Thus, a refinement of $\Delta p_{\max}$ by a certain factor causes a refinement of the whole *p* grid by the same factor. You are referred to Appendix B for some more details on the choice of the *p* grid.

For the calculation of steady state properties we employ the common strategy to evolve f(p,x,t) in time until the stationary state is reached. But, it is important to point out that for certain parameter sets of the *x* and *p* grid, a non-smooth convergence with a sudden build-up of error was observed. The problem arises due to the fact that the steady state distribution f(p,x,t→∞) may exhibit heavily oscillating regions in phase space, especially for situations where tunneling and coherence phenomena are prominent. This is exactly the case for RTDs in the resonant tunneling regime. When simulating such a situation with a too coarse grained grid, one encounters that the quantities of interest initially converge towards the true steady state values, but large errors arise as soon as the oscillations in f(p,x,t) become too short scaled to be resolved by the chosen grid. It is, therefore, mandatory to carefully inspect the time evolution of f(p,x,t) and, if possible, to check the robustness of the results upon a refinement of the grid spacing. In all simulations performed in the course of this work, the spacing of the *p* grid was the crucial factor for a smooth convergent behavior and the spacing of the *x* grid was comparatively unproblematic.

### 4.2 Convergence with respect to the domain size and grid spacing

At first we investigate the impact of the considered domain size, i.e. the size of the *p* interval and the reservoir length on the calculated quantities. We set the spacing of the *x* grid to Δx=a and for the *p* grid we choose $\Delta p_{\max}/\hbar =0.1\;{\text{nm}}^{-1}$. This combination of the *x* and *p* spacings is suited to properly resolve all of the oscillation patterns appearing in the steady state Wigner functions, at least for the presented sizes of the *x* domain (note that an increase of $L_{\mathrm{res}}$ to larger values than considered here may also require a decrease of $\Delta p_{\max}$).

**Fig. 3:**
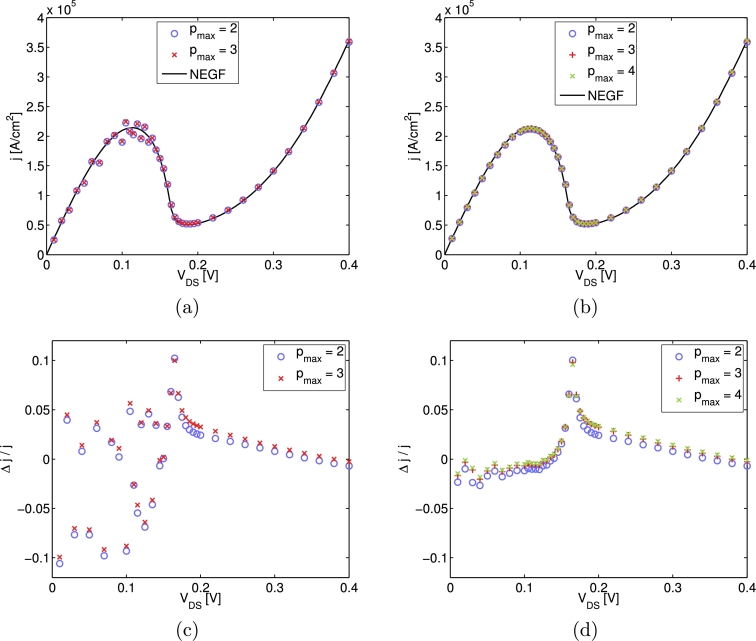
Comparison of the simulated *j*(*V*) curves for different values of the reservoir length $L_{\mathrm{res}}$ and different extensions of the *p* domain, specified by $p_{\max}/\hbar =2\;{\text{nm}}^{-1}$, $p_{\max}/\hbar =3\;{\text{nm}}^{-1}$ and $p_{\max}/\hbar =4\;{\text{nm}}^{-1}$. The results depicted on the left are for $L_{\mathrm{res}}=100\;a$ and those on the right are for $L_{\mathrm{res}}=300\;a$. The relative differences of the currents to the NEGF reference are presented in the plots on the bottom. For all of the simulations the grid spacings are chosen to Δ*x* = *a* and $\Delta p_{\max}/\hbar =0.1\;{\text{nm}}^{-1}$, so that $N_{x}=223$ (left plots) and $N_{x}=623$ (right plots), and $N_{p}\approx 1000$.

In Fig. 3, the calculated j(V) curves for simulations with different values of the reservoir length $L_{\mathrm{res}}$, as well as with different values of $p_{\max}$ are presented. A minimum value of at least $p_{\max}/\hbar =2\;{\text{nm}}^{-1}$ is chosen. For smaller values of $p_{\max}/\hbar \approx 1\;{\text{nm}}^{-1}$ one observes that the cell averages of the Wigner function at $\pm p_{\max}$ take on significant values. This indicates that the underlying assumption (see Section 3.1) that f(p,x,t) vanishes outside of the interval $\left(-p_{\max},p_{\max}\right)$, is a too severe restriction for the physical situation, for the chosen value of $p_{\max}$. As one can see from Fig. 3, the reservoir length has great impact on the accuracy of the calculated j(V) curves. Already a value of $L_{\mathrm{res}}=100\;a$ is large compared to the double barrier size of 22a (see also Fig. 2), but the influence of the boundaries on the overall device behavior is prominent. Increasing the reservoir length to $L_{\mathrm{res}}=200\;a$ (not plotted) lessens this influence and for $L_{\mathrm{res}}=300\;a$ a fairly good agreement between the Wigner function and the NEGF calculations is observed in the whole voltage range (0V,0.4 V). Taking the minor differences in the results for $p_{\max}/\hbar =3\;{\text{nm}}^{-1}$ and $p_{\max}/\hbar =4\;{\text{nm}}^{-1}$ into account, we conclude that a size of the (p,x) domain determined by $L_{\mathrm{res}}=300\;a$ and $p_{\max}/\hbar =3\;{\text{nm}}^{-1}$ is a reasonable choice for the considered physical problem. A convergence study for the particle density n(x) does not give additional insight and is not presented here. A similar dependence on $L_{\mathrm{res}}$ as for the case of the j(V)-curves was found, whereby the magnitude of the errors in n(x) is much smaller and already results for $L_{\mathrm{res}}=100\;a$ agree fairly well with the NEGF reference.

We now focus on the question of the convergence of the calculated quantities with respect to the grid spacing. In particular we reduce Δ*x* and consider one fixed but adjusted value of $\Delta p_{\max}$. Once the Wigner function is already well-resolved with respect to the momentum variable, a further decrease of the *p* spacing has only minor influence on the calculated quantities.

**Fig. 4:**
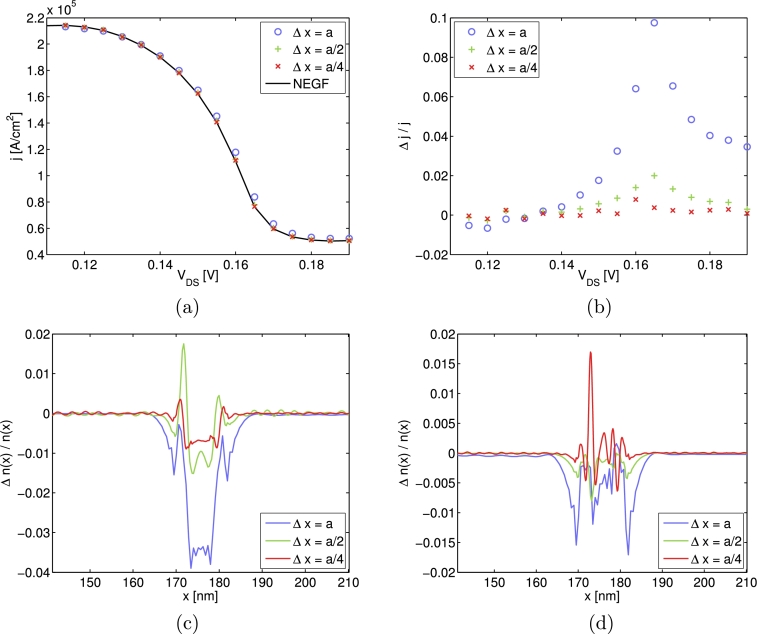
Plot (a) depicts the *j*(*V*) curve for the NDR-region, (b) the relative difference of the *j*(*V*) values to the NEGF solution and (c) and (d) the relative difference in particle density for $V_{\mathrm{DS}}=0.115\;\text{V}$ and $V_{\mathrm{DS}}=0.185\;V$, respectively. The results are obtained by using $\Delta p_{\max}/\hbar =0.075\;{\text{nm}}^{-1}$ and three different spacings $\Delta x=a,\frac{a}{2},\frac{a}{4}$. The size of the (*p*,*x*) domain is the same in all three simulations and determined by $L_{\mathrm{res}}=300\;a$ and $p_{\max}/\hbar =3\;{\text{nm}}^{-1}$. The number of grid points is $N_{x}=623,1245,2489$ and $N_{p}\approx 1400$.

In Fig. 4, the results for three different spacings Δx=a, $\Delta x=\frac{a}{2}$ and $\Delta x=\frac{a}{4}$ are compared. For the particle densities n(x), the relative errors for the two voltages $V_{\mathrm{DS}}=0.115\;\text{V}$ and $V_{\mathrm{DS}}=0.185\;\text{V}$ are shown in Fig. 4(c), (d). For j(V), only the region of negative differential resistance (NDR) is plotted due to its particular importance for device applications. It is obvious to see a monotonic decrease of the error in the current density with decreasing values of Δ*x* and a very good agreement between the NEGF reference and the WTE calculations for the case $\Delta x=\frac{a}{4}$, with a relative error |Δj/j| below one percent. This clearly demonstrates the ability of the developed algorithm to produce very accurate results and furthermore, the convergence of the calculated quantities when refining the grid parameters.

### 4.3 Wigner functions for the steady state

After these preliminary convergence studies we now focus on more physical aspects and present steady state Wigner functions for which we know that the results are well-converged. Fig. 3 displays the characteristic S-shaped j(V) curve of a RTD including a negative differential resistance (NDR) [7]. The peak in j(V) at $V_{\mathrm{DS}}\approx 0.115\;\text{V}$ is taken on when resonant tunneling is most prominent. The resonant tunneling decreases in the NDR-region until one enters the regime of conventional tunneling at the valley, $V_{\mathrm{DS}}\approx 0.185\;\text{V}$, and for higher voltages.

**Fig. 5:**
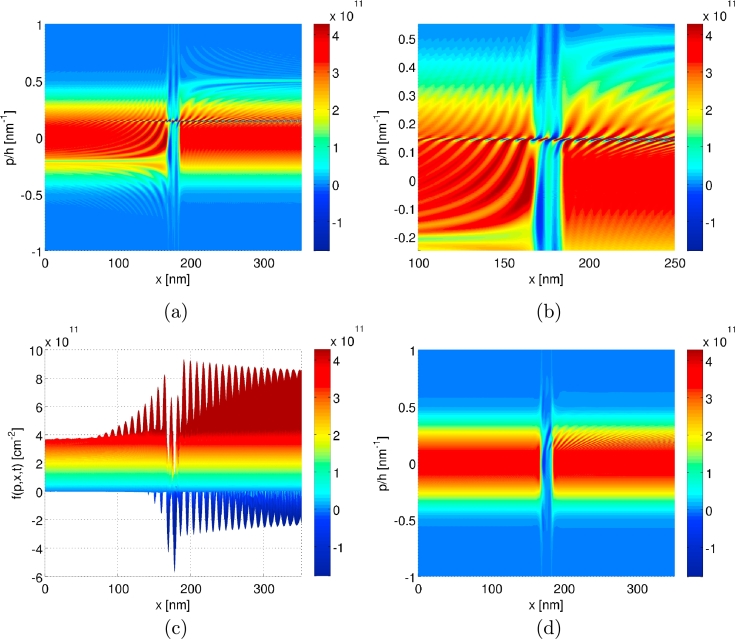
Illustrations of the steady state solutions of *f*(*p*,*x*,*t*) obtained by using the parameters $\Delta x=\frac{a}{2}$, $\Delta p_{\max}/\hbar =0.05\;{\text{nm}}^{-1}$, $L_{\mathrm{res}}=300\;a$ and $p_{\max}/\hbar =3\;{\text{nm}}^{-1}$ for two different bias voltages. Depicted in (a), (b), (c) are the solutions for $V_{\mathrm{DS}}=0.115\;V$ and in (d) the one for $V_{\mathrm{DS}}=0.185\;\text{V}$. The number of grid points for the two cases are $N_{x}=1245$, $N_{p}=2151$ and $N_{x}=1245$, $N_{p}=2048$ for $V_{\mathrm{DS}}=0.115\;\text{V}$ and $V_{\mathrm{DS}}=0.185\;\text{V}$, respectively.

To see how the two physically dissimilar regimes manifest itself in phase space, Fig. 5 displays steady state Wigner functions for the voltages $V_{\mathrm{DS}}=0.115\;\text{V}$ and $V_{\mathrm{DS}}=0.185\;\text{V}$. In the conventional tunneling regime (Fig. 5(d)), the Wigner function f(p,x,t) is negative-valued only in small regions of the phase space and takes on a rather smooth shape, close to a classical Boltzmann distribution. In the resonant tunneling regime (Fig. 5(a)–(c)), on the other hand, the emergence of heavily oscillating regions in phase space is apparent. On the one hand, rather long-scaled oscillations are present throughout large parts of the phase space and on the other hand, a very sharp and large-valued stripe of oscillations builds up around $p/\hbar \approx 0.15\;{\text{nm}}^{-1}$. The detailed phase space dynamics are involved but one can see in the reflected part of the distribution on the left-hand side that momenta of approximately $p/\hbar \approx -0.2\;{\text{nm}}^{-1}$ are extenuated, corresponding to the part for which a resonant tunneling process is accessible. On the right-hand side, one can see the outgoing, accelerated beam at $p/\hbar \approx 0.5\;{\text{nm}}^{-1}$. The sharp stripe of oscillations at $k_{osc}=p/\hbar \approx 0.15\;{\text{nm}}^{-1}$ is closely related to the resonant tunneling process itself and to the coherence between the reflected and transmitted part at $k_{r}=p/\hbar \approx -0.2\;{\text{nm}}^{-1}$ and $k_{t}=p/\hbar \approx 0.5\;{\text{nm}}^{-1}$, respectively. At least this is what we conclude after analyzing various simulations and performing analytical calculations for simple tunneling situations. When solving for the Wigner function of a single, unbiased tunneling barrier and for the case of incoming plane waves, singular terms of the form δ(p) and 1/p together with oscillating prefactors with respect to *x* emerge (see also [51]). A superposition of plane waves with different wavenumbers $\left|k^{\prime }|\neq |k^{\prime \prime }\right|$ causes a change of the form $\delta (p)\to \delta (p-\hbar (k^{\prime }+k^{\prime \prime })/2)$. Such a behavior is consistent with $k_{osc}=(k_{r}+k_{t})/2$. The question may arise why the simulations reveal a sharply peaked and heavily oscillating behavior of f(p,x,t) but no singular terms. To our understanding this is a consequence of the non-zero bias voltage combined with a continuous potential. Tests for the present RTD and a bias of $V_{\mathrm{DS}}=0\;\text{V}$ were performed, with the result that sharp oscillations arose in the central region at $p/\hbar \approx 0\;{\text{nm}}^{-1}$ which could not be properly resolved upon decreasing the *p* spacing. We thus believe that it is in general advisable to consider only non-zero bias voltages and preferably situations with a continuous V(x). Due to the presence of the singular terms δ(p) and 1/p for the case of unbiased barriers, we cannot provide reliable simulations with the present algorithm to the situations discussed in [27,28]. We can only report on the observation that unphysical solutions were encountered indeed, but solely in the case of under-resolved momentum grids.

From the knowledge of the detailed shape of the steady state Wigner functions in phase space it is possible to understand the particular dependence of the j(V)-curves on the reservoir length $L_{\mathrm{res}}$. As in the case of classical transport theory, the application of the inflow/outflow boundary conditions is only an appropriate approximation if, in the vicinity of the boundaries, the gradients of f(p,x,t) with respect to *x* are negligible. It corresponds to the requirement that the action of the pseudo-differential operator close to the boundaries and thereafter, of course, is not significant. From Fig. 3 it is obvious to see that the low-bias regime of $V_{\mathrm{DS}}≲0.16\;\text{V}$, where resonant tunneling is prominent, converges much slower with respect to $L_{\mathrm{res}}$ than the high-bias regime of $V_{\mathrm{DS}}≳0.16\;\text{V}$, where conventional tunneling dominates. For the case of the conventional tunneling regime, one can note from Fig. 5(d) that f(p,x,t) exhibits only comparatively weak oscillations outside the region of the double barrier. In contrast, the results in Fig. 5(a) are characterized by strong and long-ranged oscillations of f(p,x,t) in the resonant tunneling regime. Clearly, the gradients of f(p,x,t) with respect to *x* at position x≈100a are much smaller in the former case than in the latter one.

### 4.4 Transient response simulation

The Wigner function formalism gives direct access to time-resolved quantities and enables one to address time-dependent situations. In the following we present results for a simple example, namely the large-signal transient response of a RTD [8]. Of particular interest for this are the two dissimilar situations at $V_{\mathrm{DS}}=0.115\;\text{V}$ and $V_{\mathrm{DS}}=0.185\;\text{V}$, corresponding to the peak and to the valley in the j(V) curves, respectively. For the simulations termed peak-to-valley we start from the steady state Wigner function for the peak voltage and consider an abrupt switching in bias voltage at t=0 fs, and for the case valley-to-peak the same is done in the opposite way.

**Fig. 6:**
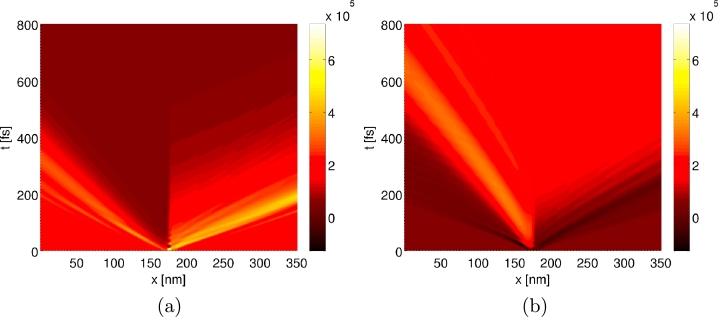
Time evolution of the current density *j*(*x*,*t*) for the transient response simulations, in (a) for peak-to-valley and in (b) for valley-to-peak. The parameters for the simulations are Δ*x* = *a*, $\Delta p_{\max}/\hbar =0.1\;{\text{nm}}^{-1}$, $L_{\mathrm{res}}=300\;a$ and $p_{\max}/\hbar =3\;{\text{nm}}^{-1}$, i.e. $N_{x}=623$ and $N_{p}=1076$. In both cases the same *p* grid is used, namely the one optimized for the peak voltage.

**Fig. 7:**
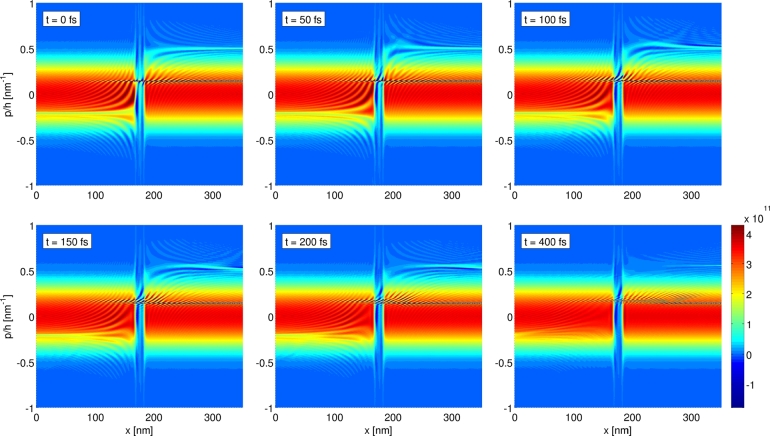
Time evolution of *f*(*p*,*x*,*t*) in phase space for the peak-to-valley case. For the simulation parameters see Fig. 6.

**Fig. 8:**
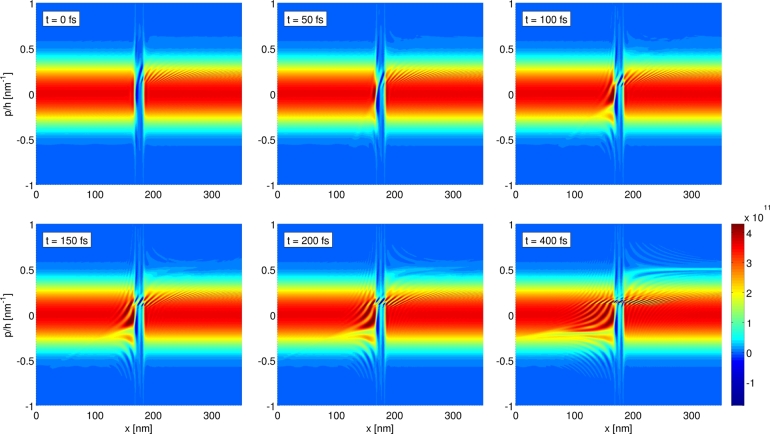
Time evolution of *f*(*p*,*x*,*t*) in phase space for the valley-to-peak case. For the simulation parameters see Fig. 6.

To examine the dynamics in detail, Figs. 6–8 depict the time evolution of the current density j(x,t) as well as of the Wigner function. For the case of the peak-to-valley simulation, it is apparent to see from Fig. 6(a) an initial rise of the current density in the region of the double barrier, which then propagates to the contact on the right-hand side. This corresponds to the part of the electron distribution which occupied the well state at t=0 fs and is then accelerated to higher momenta by the increased bias voltage, see also Fig. 7 at $p/\hbar \approx 0.5\;{\text{nm}}^{-1}$ and x≳200 nm. Overall, the relaxation of j(x,t) to the new stationary value is much faster on the upwind side (left-hand side) of the barrier than on the downwind side. Fig. 6(b) depicts the time evolution of j(x,t) for the case of switching from valley to peak. Similar to the previous case, the time scale for the change in current density is shorter on the upwind side than on the downwind one. The abrupt rise in current density on the upwind side can be accounted to the reduction in reflection of electrons on the left-hand side of the barriers, as a result of the resonant tunneling of some of the electrons through the well state. In phase space, this effect is visible as an extenuation of the Wigner distribution on the upwind side at the corresponding momenta, see Fig. 8 at $p/\hbar \approx -0.2\;{\text{nm}}^{-1}$ and x≲170 nm.

The time-resolved phase space plots of f(p,x,t) in Fig. 8 allow one to investigate the detailed dynamics of the advent of the sharp stripe of oscillations at $p/\hbar \approx 0.15\;{\text{nm}}^{-1}$. In the beginning, the oscillations arise in the center at x≈175 nm, rather parallel to the *p* axis and with a long-scaled modulation, before spreading out to larger values of *x* together with becoming more short-scaled. In the course of this evolution the oscillations turn more parallel to the *x* axis in sort of a shear movement. As a result, a very short-scaled modulation of f(p,x,t) with respect to *p* is formed, which requires an extremely fine *p* grid to resolve the steady state Wigner function properly ($\Delta p_{\min}/\hbar \approx 4\times 10^{-4}\;{\text{nm}}^{-1}$). Upon increasing $L_{\mathrm{res}}$, the required resolution with respect to *p* increases further. We believe that this effect is most prominent for the ballistic case as considered here, and that one can expect a damping of the oscillations as soon as dissipative mechanisms, such as electron–phonon scattering are included.

## 5 Conclusion and outlook

In this work, a novel numerical scheme for the deterministic solution of the Wigner transport equation has been presented. The central aspect of the method is to allow for a highly flexible and adaptive choice of the simulation domain. A detailed study of convergence is given by comparing the WTE computations to a reference solution which we obtained with the NEGF method. The results clearly demonstrate the convergence of the algorithm and a quasi-exact agreement between the WTE and the NEGF calculations could be obtained. As indicated already in other studies [44], the application of the inflow/outflow boundary conditions may yield physically valid and correct results, provided a sufficiently large distance between the active device region and the boundaries of the simulation domain is included. For large enough computational domains, a monotonic convergence of the current–voltage characteristics towards the NEGF reference could be achieved upon refining the grid spacing, with a minimal relative error below one percent. Subsequent to the convergence study, steady state Wigner functions for the RTD operating in the resonant and in the conventional tunneling regime have been compared and discussed. The two physically dissimilar regimes manifest itself in phase space by drastically different Wigner functions. In the resonant tunneling case, a sharp and large-valued stripe of oscillations has been discovered, accounting for the coherent superposition of reflected and transmitted states. In the final part of this work, a transient response simulation has been considered together with investigating the dynamics of the current density and the Wigner function.

Possible extensions of the method and further investigations are manifold. Since RTDs are meant to operate in the THz regime, fully time-dependent calculations are of interest. As investigated already thoroughly by other groups, a self-consistent solution of the Wigner–Poisson system including scattering mechanisms is important for a realistic description of transport at room temperature and could be also combined, of course, with the approach presented here.

## Figures and Tables

**Fig. B.9:**
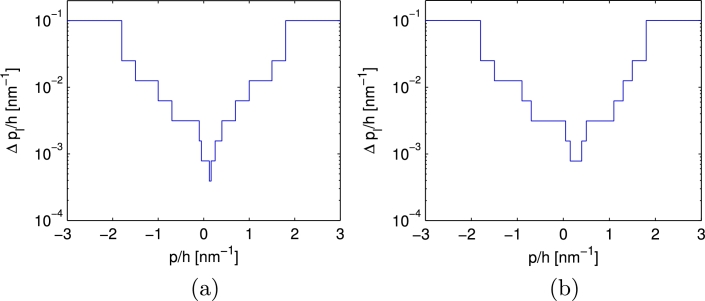
Illustration of the dependence of the grid spacing $\Delta p_{l}$ on *p* and on the applied bias $V_{\mathrm{DS}}$. The plot in (a) is for $V_{\mathrm{DS}}=0.115\;\text{V}$ and in (b) for $V_{\mathrm{DS}}=0.3\;\text{V}$, with $\Delta p_{\max}/\hbar =0.1\;{\text{nm}}^{-1}$ and $p_{\max}/\hbar =3\;{\text{nm}}^{-1}$.

## Appendix A Calculational details for the discretization of the pseudo-differential operator

In the following we outline details for the calculation of the matrix elements $D_{m,i,j}^{F}$ and $D_{m,i,j}^{\sigma }$ in Eq. (45), starting from Eq. (42). For the sake of clarity we consider at first a single potential segment to evaluate the integrals and combine the resulting equations thereafter in order to arrive at the final expressions for the matrix elements. For this purpose, we choose a time-independent potential which is linear in the interval $\left(x_{a},x_{b}\right)$ and zero elsewhere. The constant part at x=0 is denoted by $v^{0}$ and the slope by $v^{1}$. By doing so Eq. (42) simplifies to

(A.1) $$\partial_{t}F_{m,j}(t)=\frac{1}{\Delta p_{m}\Delta x}\frac{2q}{\hbar }\int_{x_{j-1/2}}^{x_{j+1/2}}\int_{2(x_{a}-x)}^{2(x_{b}-x)}\left[v^{0}+v^{1}\left(x+\frac{\eta^{\prime }}{2}\right)\right]K_{m,j}\left(\eta^{\prime },t\right)d\eta^{\prime }dx.$$

When inserting Eq. (43) for $K_{m,j}(\eta^{\prime },t)$ and Eq. (44) for the Fourier transform $\overset{˜}{f}(\eta^{\prime },x_{j},t)$, whereby the integration with respect to $p^{\prime }$ is readily done, we obtain for the matrix elements

(A.2) $$D_{m,i,j}^{F}=\frac{q}{\pi \Delta p_{m}\Delta x}\int_{x_{j-1/2}}^{x_{j+1/2}}\int_{2(x_{a}-x)}^{2(x_{b}-x)}\left[v^{0}+v^{1}\left(x+\frac{\eta^{\prime }}{2}\right)\right]ℜ\left\{\frac{i}{\eta^{\prime \;2}}\left[\exp\left(-i\frac{p_{i+1/2}}{\hbar }\eta^{\prime }\right)-\exp\left(-i\frac{p_{i-1/2}}{\hbar }\eta^{\prime }\right)\right]\left[\exp\left(i\frac{p_{m+1/2}}{\hbar }\eta^{\prime }\right)-\exp\left(i\frac{p_{m-1/2}}{\hbar }\eta^{\prime }\right)\right]\right\}d\eta^{\prime }dx,\;m=2,\ldots ,N_{p}-1,\;\;j=1,\ldots ,N_{x},$$

and

(A.3) $$D_{m,i,j}^{\sigma }=\frac{q}{\pi \Delta p_{m}\Delta x}\int_{x_{j-1/2}}^{x_{j+1/2}}\int_{2(x_{a}-x)}^{2(x_{b}-x)}\left[v^{0}+v^{1}\left(x+\frac{\eta^{\prime }}{2}\right)\right]ℜ\left\{\left\{\frac{\Delta p_{i}}{2}\frac{i}{\eta^{\prime \;2}}\left[\exp\left(-i\frac{p_{i+1/2}}{\hbar }\eta^{\prime }\right)+\exp\left(-i\frac{p_{i-1/2}}{\hbar }\eta^{\prime }\right)\right]+\frac{\hbar }{\eta^{\prime \;3}}\left[\exp\left(-i\frac{p_{i+1/2}}{\hbar }\eta^{\prime }\right)-\exp\left(-i\frac{p_{i-1/2}}{\hbar }\eta^{\prime }\right)\right]\right\}\left[\exp\left(i\frac{p_{m+1/2}}{\hbar }\eta^{\prime }\right)-\exp\left(i\frac{p_{m-1/2}}{\hbar }\eta^{\prime }\right)\right]\right\}d\eta^{\prime }dx,\;m=2,\ldots ,N_{p}-1,\;j=1,\ldots ,N_{x},$$

where $\Delta p_{i}=p_{i+1/2}-p_{i-1/2}$. For the boundary terms m=1 and $m=N_{p}$ similar expressions are readily written down.

In the course of calculating the integrals in Eqs. (A.2) and (A.3) one encounters sine and cosine integrals. For their standard definitions see for instance [20,52,53]. In practice we employed library routines which use a combination of a series expansion and complex continued fraction with a certain crossover point [20]. The most basic cosine integral we will encounter in the following is

(A.4) $$\int_{kd_{1}}^{kd_{2}}\frac{1}{t}\cos(t)dt=\int_{\left|kd_{1}\right|}^{\left|kd_{2}\right|}\frac{1}{t}\cos(t)dt=\mathrm{Ci}\left(|kd_{2}|\right)-\mathrm{Ci}\left(|kd_{1}|\right),$$

where *k* labels some wavenumber and $d_{\alpha }$ some distance. The same expression for the sine integral is given by

(A.5) $$\int_{kd_{1}}^{kd_{2}}\frac{1}{t}\sin(t)dt=\mathrm{Si}(kd_{2})-\mathrm{Si}(kd_{1}).$$

For the averaging process we additionally need the primitive integrals [52,53]

(A.6) ∫Ci(z)dz=zCi(z)−sin⁡(z),∫Si(z)dz=zSi(z)+cos⁡(z).

### A.1 Constant part of the polynomial for the Wigner function

Here, we focus on the calculation of the matrix elements $D_{m,i,j}^{F}$, given by Eq. (A.2). It is convenient to define

(A.7) $$I_{m+1/2,i+1/2,j}^{F}=v^{0}I_{m+1/2,i+1/2,j}^{F,v^{0}}+v^{1}I_{m+1/2,i+1/2,j}^{F,v^{1}},$$

with

(A.8) $$I_{m+1/2,i+1/2,j}^{F,v^{0}}=-\int_{x_{j-1/2}}^{x_{j+1/2}}\int_{2(x_{a}-x)}^{2(x_{b}-x)}\frac{1}{\eta^{\prime \;2}}\sin\left(\frac{p_{m+1/2}-p_{i+1/2}}{\hbar }\eta^{\prime }\right)d\eta^{\prime }dx,$$

and

(A.9) $$I_{m+1/2,i+1/2,j}^{F,v^{1}}=-\int_{x_{j-1/2}}^{x_{j+1/2}}\int_{2(x_{a}-x)}^{2(x_{b}-x)}\left(x+\frac{\eta^{\prime }}{2}\right)\frac{1}{\eta^{\prime \;2}}\sin\left(\frac{p_{m+1/2}-p_{i+1/2}}{\hbar }\eta^{\prime }\right)d\eta^{\prime }dx,$$

so that $D_{m,i,j}^{F}$ is given by the expressions stated in Eq. (46).

To evaluate $I_{m+1/2,i+1/2,j}^{F,v^{0}}$, one can perform the integration with respect to $\eta^{\prime }$ by taking Eq. (A.4) into account and thereafter, make use of Eqs. (A.5) and (A.6) to finally obtain

(A.10) $$I_{m+1/2,i+1/2,j}^{F,v^{0}}=T^{F,v^{0}}(u){|}_{C_{m,i,j}^{x_{b}}}-T^{F,v^{0}}(u){|}_{C_{m,i,j}^{x_{a}}},$$

with

(A.11) $$T^{F,v^{0}}(u)=\frac{1}{2}\left[u\mathrm{Ci}\left(|u|\right)-\mathrm{Si}(u)-\sin(u)\right].$$

Here, the abbreviations for the intervals

(A.12) $$C_{m,i,j}^{x_{a}}=\left(2k_{m,i}(x_{a}-x_{j-1/2}),2k_{m,i}(x_{a}-x_{j+1/2})\right),\;C_{m,i,j}^{x_{b}}=\left(2k_{m,i}(x_{b}-x_{j-1/2}),2k_{m,i}(x_{b}-x_{j+1/2})\right),$$

are used together with $k_{m,i}$ as defined in Eq. (51).

For $I_{m+1/2,i+1/2,j}^{F,v^{1}}$ we follow analogous steps and find under consideration of Eqs. (A.4)–(A.6)

(A.13) $$I_{m+1/2,i+1/2,j}^{F,v^{1}}=x_{b}T^{F,v^{0}}(u){|}_{C_{m,i,j}^{x_{b}}}-x_{a}T^{F,v^{0}}(u){|}_{C_{m,i,j}^{x_{a}}}+\frac{1}{k_{m,i}}\left[T^{F,v^{1}}(u){|}_{C_{m,i,j}^{x_{b}}}-T^{F,v^{1}}(u){|}_{C_{m,i,j}^{x_{a}}}\right],$$

with $T^{F,v^{0}}(u)$ defined in Eq. (A.11) and

(A.14) $$T^{F,v^{1}}(u)=\frac{1}{8}\left[-u^{2}\mathrm{Ci}\left(|u|\right)+2u\mathrm{Si}(u)+u\sin(u)+\cos(u)\right].$$

### A.2 Linear part of the polynomial for the Wigner function

For the calculation of the matrix elements $D_{m,i,j}^{\sigma }$ (see Eq. (A.3)) we define

(A.15) $$I_{m+1/2,i^{\pm },j}^{\sigma }=v^{0}I_{m+1/2,i^{\pm },j}^{\sigma ,v^{0}}+v^{1}I_{m+1/2,i^{\pm },j}^{\sigma ,v^{1}},$$

with

(A.16) $$I_{m+1/2,i^{+},j}^{\sigma ,v^{0}}=\int_{x_{j-1/2}}^{x_{j+1/2}}\int_{2(x_{a}-x)}^{2(x_{b}-x)}\left[-\frac{\Delta p_{i}}{2}\frac{1}{\eta^{\prime \;2}}\sin\left(\frac{p_{m+1/2}-p_{i+1/2}}{\hbar }\eta^{\prime }\right)+\frac{\hbar }{\eta^{\prime \;3}}\cos\left(\frac{p_{m+1/2}-p_{i+1/2}}{\hbar }\eta^{\prime }\right)\right]d\eta^{\prime }dx$$

and

(A.17) $$I_{m+1/2,i^{-},j}^{\sigma ,v^{0}}=-\int_{x_{j-1/2}}^{x_{j+1/2}}\int_{2(x_{a}-x)}^{2(x_{b}-x)}\left[-\frac{\Delta p_{i}}{2}\frac{1}{\eta^{\prime \;2}}\sin\left(\frac{p_{m+1/2}-p_{i-1/2}}{\hbar }\eta^{\prime }\right)-\frac{\hbar }{\eta^{\prime \;3}}\cos\left(\frac{p_{m+1/2}-p_{i-1/2}}{\hbar }\eta^{\prime }\right)\right]d\eta^{\prime }dx,$$

as well as

(A.18) $$I_{m+1/2,i^{+},j}^{\sigma ,v^{1}}=\int_{x_{j-1/2}}^{x_{j+1/2}}\int_{2(x_{a}-x)}^{2(x_{b}-x)}\left(x+\frac{\eta^{\prime }}{2}\right)\left[-\frac{\Delta p_{i}}{2}\frac{1}{\eta^{\prime \;2}}\sin\left(\frac{p_{m+1/2}-p_{i+1/2}}{\hbar }\eta^{\prime }\right)+\frac{\hbar }{\eta^{\prime \;3}}\cos\left(\frac{p_{m+1/2}-p_{i+1/2}}{\hbar }\eta^{\prime }\right)\right]d\eta^{\prime }dx$$

and the analogous expression for $I_{m+1/2,i^{-},j}^{\sigma ,v^{1}}$. The matrix elements $D_{m,i,j}^{\sigma }$ are then given by Eq. (47).

To calculate $I_{m+1/2,i^{\pm },j}^{\sigma ,v^{0}}$ we first integrate with respect to $\eta^{\prime }$, make use of Eqs. (A.4)–(A.6) as well as of the result for $I_{m+1/2,i+1/2,j}^{F,v^{0}}$, see Eqs. (A.8) and (A.10), to finally arrive at

(A.19) $$I_{m+1/2,i^{+},j}^{\sigma ,v^{0}}=\left(\frac{p_{m+1/2}+p_{i+1/2}}{2}-p_{i}\right)\left[T^{F,v^{0}}(u){|}_{C_{m,i,j}^{x_{b}}}-T^{F,v^{0}}(u){|}_{C_{m,i,j}^{x_{a}}}\right]-(p_{m+1/2}-p_{i+1/2})\left[T^{\sigma ,v^{0}}(u){|}_{C_{m,i,j}^{x_{b}}}-T^{\sigma ,v^{0}}(u){|}_{C_{m,i,j}^{x_{a}}}\right],$$

and

(A.20) $$I_{m+1/2,i^{-},j}^{\sigma ,v^{0}}=\left(\frac{p_{m+1/2}+p_{i-1/2}}{2}-p_{i}\right)\left[T^{F,v^{0}}(u){|}_{C_{m,i-1,j}^{x_{b}}}-T^{F,v^{0}}(u){|}_{C_{m,i-1,j}^{x_{a}}}\right]-(p_{m+1/2}-p_{i-1/2})\left[T^{\sigma ,v^{0}}(u){|}_{C_{m,i-1,j}^{x_{b}}}-T^{\sigma ,v^{0}}(u){|}_{C_{m,i-1,j}^{x_{a}}}\right],$$

expressed in terms of $T^{F,v^{0}}(u)$, see Eq. (A.11), as well as

(A.21) $$T^{\sigma ,v^{0}}(u)=\frac{1}{4}\left[\frac{1}{u}\cos(u)+\mathrm{Si}(u)\right].$$

In an analogous manner we perform the integrations in $I_{m+1/2,i^{\pm },j}^{\sigma ,v^{1}}$ with the help of Eqs. (A.4)–(A.6) and the result for $I_{m+1/2,i+1/2,j}^{F,v^{1}}$, see Eqs. (A.9) and (A.13). On the whole we find after some lengthy calculations

(A.22) $$I_{m+1/2,i^{+},j}^{\sigma ,v^{1}}=\left(\frac{p_{m+1/2}+p_{i+1/2}}{2}-p_{i}\right)\left\{x_{b}T^{F,v^{0}}(u){|}_{C_{m,i,j}^{x_{b}}}-x_{a}T^{F,v^{0}}(u){|}_{C_{m,i,j}^{x_{a}}}+\frac{1}{k_{m,i}}\left[T^{F,v^{1}}(u){|}_{C_{m,i,j}^{x_{b}}}-T^{F,v^{1}}(u){|}_{C_{m,i,j}^{x_{a}}}\right]\right\}-\hbar k_{m,i}\left[x_{b}T^{\sigma ,v^{0}}(u){|}_{C_{m,i,j}^{x_{b}}}-x_{a}T^{\sigma ,v^{0}}(u){|}_{C_{m,i,j}^{x_{a}}}\right]+\hbar \left[T^{\sigma ,v^{1}}(u){|}_{C_{m,i,j}^{x_{b}}}-T^{\sigma ,v^{1}}(u){|}_{C_{m,i,j}^{x_{a}}}\right],$$

and

(A.23) $$I_{m+1/2,i^{-},j}^{\sigma ,v^{1}}=\left(\frac{p_{m+1/2}+p_{i-1/2}}{2}-p_{i}\right)\left\{x_{b}T^{F,v^{0}}(u){|}_{C_{m,i-1,j}^{x_{b}}}-x_{a}T^{F,v^{0}}(u){|}_{C_{m,i-1,j}^{x_{a}}}+\frac{1}{k_{m,i-1}}\left[T^{F,v^{1}}(u){|}_{C_{m,i-1,j}^{x_{b}}}-T^{F,v^{1}}(u){|}_{C_{m,i-1,j}^{x_{a}}}\right]\right\}-\hbar k_{m,i-1}\left[x_{b}T^{\sigma ,v^{0}}(u){|}_{C_{m,i-1,j}^{x_{b}}}-x_{a}T^{\sigma ,v^{0}}(u){|}_{C_{m,i-1,j}^{x_{a}}}\right]+\hbar \left[T^{\sigma ,v^{1}}(u){|}_{C_{m,i-1,j}^{x_{b}}}-T^{\sigma ,v^{1}}(u){|}_{C_{m,i-1,j}^{x_{a}}}\right]$$

with the definitions Eqs. (A.11), (A.14) and (A.21) as well as

(A.24) $$T^{\sigma ,v^{1}}(u)=\frac{1}{8}\left[\mathrm{Ci}(|u|)+u\mathrm{Si}(u)+\cos(u)\right].$$

### A.3 Diagonal elements and compound potential shapes

For computational reasons it is important to analyze the behavior of the terms $I_{m+1/2,i+1/2,j}^{F,v^{0/1}}$ and $I_{m+1/2,i^{\pm },j}^{\sigma ,v^{0/1}}$ for small arguments since singular functions are present in some cases. In particular we examine the case $k_{m,m}=0$, needed for the diagonal terms. With the definition of the sine and cosine integrals Eqs. (A.4) and (A.5) we find the following limits (A.25)

$$\lim_{u\to 0}T^{F,v^{0}}(u)=0,\;\lim_{k\to 0}kT^{\sigma ,v^{0}}(u){|}_{kd_{1}}^{kd_{2}}=\frac{1}{4}\left[\frac{1}{d_{2}}-\frac{1}{d_{1}}\right],$$

$$\lim_{k\to 0}\frac{1}{k}T^{F,v^{1}}(u){|}_{kd_{1}}^{kd_{2}}=0,\;\lim_{k\to 0}T^{\sigma ,v^{1}}(u){|}_{kd_{1}}^{kd_{2}}=\frac{1}{8}\left[\ln|d_{2}|-\ln|d_{1}|\right],$$

 when using *k* instead of $k_{m,i}$ together with $d_{1}$ and $d_{2}$ labeling two distances. As a result, $I_{m+1/2,m+1/2,j}^{F,v^{0/1}}=0$ and $I_{m+1/2,m^{\pm },j}^{\sigma ,v^{0/1}}$ is in general non-zero.

Up to now we considered the simple case of a single potential segment, as specified in Eq. (A.1). The piecewise linear approximation of a potential V(x,t) in Eqs. (39) and (40) is a sum of such segments. Due to the linearity of the pseudo-differential operator it is obvious that the matrix elements for such a compound potential shape are simply given as a sum of the contributions from the individual parts. To arrive at the final equation stated in Eq. (48) we still need to show how a bias–voltage enters the equations. For this we choose a potential V(x,t) which is given by the sum of two parts:

(A.26) $$V(x)=V_{\mathrm{DS}}H\left(x-x_{c}^{V}\right)H\left(x_{0}^{V}-x\right)+V_{\mathrm{DS}}\frac{x_{L}^{V}-x}{x_{L}^{V}-x_{0}^{V}}H\left(x-x_{0}^{V}\right)H\left(x_{L}^{V}-x\right),\;x\in R,$$

whereby *H* denotes the Heaviside step function, the positions $x_{0}^{V}$ and $x_{L}^{V}$ label the device endpoints and $x_{c}^{V}$ is some point inside the contact on the left-hand side which we will let go to −∞. The drain-source voltage is of magnitude $V_{\mathrm{DS}}$. When summing up the terms involved in Eqs. (A.7) and (A.15) resulting from the two parts, we find

(A.27) $$I_{m+1/2,i+1/2,j}^{F}=-V_{\mathrm{DS}}\left\{T^{F,v^{0}}(u){|}_{C_{m,i,j}^{x_{c}^{V}}}+\frac{1}{x_{L}^{V}-x_{0}^{V}}\frac{1}{k_{m,i}}\left[T^{F,v^{1}}(u){|}_{C_{m,i,j}^{x_{L}^{V}}}-T^{F,v^{1}}(u){|}_{C_{m,i,j}^{x_{0}^{V}}}\right]\right\},$$

(A.28) $$I_{m+1/2,i^{+},j}^{\sigma }=-V_{\mathrm{DS}}\left\{\left(\frac{p_{m+1/2}+p_{i+1/2}}{2}-p_{i}\right)T^{F,v^{0}}(u){|}_{C_{m,i,j}^{x_{c}^{V}}}-\hbar k_{m,i}T^{\sigma ,v^{0}}(u){|}_{C_{m,i,j}^{x_{c}^{V}}}\right\}-V_{\mathrm{DS}}\frac{1}{x_{L}^{V}-x_{0}^{V}}\left\{\hbar \left[T^{\sigma ,v^{1}}(u){|}_{C_{m,i,j}^{x_{L}^{V}}}-T^{\sigma ,v^{1}}(u){|}_{C_{m,i,j}^{x_{0}^{V}}}\right]+\left(\frac{p_{m+1/2}+p_{i+1/2}}{2}-p_{i}\right)\frac{1}{k_{m,i}}\left[T^{F,v^{1}}(u){|}_{C_{m,i,j}^{x_{L}^{V}}}-T^{F,v^{1}}(u){|}_{C_{m,i,j}^{x_{0}^{V}}}\right]\right\},$$

whereby all other terms with index $v^{0}$ drop out of the equations. From the definitions of the sine and cosine integrals Eqs. (A.4) and (A.5) together with the asymptotic limit [53]

(A.29) $$\lim_{x\to \infty }\mathrm{Si}(x)=\frac{\pi }{2},$$

we obtain that the contributions from the potential endpoint $x_{c}^{V}$ vanish as $x_{c}^{V}\to -\infty$: (A.30)

$$\lim_{x_{c}^{V}\to -\infty }T^{F,v^{0}}(u){|}_{C_{m,i,j}^{x_{c}^{V}}}=0,$$

$$\lim_{x_{c}^{V}\to -\infty }T^{\sigma ,v^{0}}(u){|}_{C_{m,i,j}^{x_{c}^{V}}}=0.$$

 Therefore, only terms with index $v^{1}$ are left in Eqs. (A.27) and (A.28), which is the case for any continuous potential shape. The expressions for more complicated potentials with multiple segments are obtained in the same manner and one can directly deduce the final result Eq. (48) from the calculations outlined here.

## Appendix B Spacing of the momentum grid and computational aspects

To briefly discuss the high demands on the resolution of the momentum grid, Fig. B.9 depicts a typical discretization used in practice, where the individual spacings $\Delta p_{l}$ are plotted for the different *p* values inside the simulated domain. In order to be able to resolve the heavily oscillating region of f(p,x,t) around p⪆0 properly, multiple refinement steps are used and adapted to the particular bias voltage. As one can see, the required resolution in the central region is more than two orders of magnitude higher than in the outermost part. An equidistant grid with the same resolution would require approximately $1.5\times 10^{4}$ grid points, which exceeds the capacities of standard computing resources.

From a numerical point of view, it is advisable to choose all the grid spacings $\Delta p_{l}$ as some multiple integer of a minimum spacing. By doing so, only a very limited number of different terms $u=k_{m,i}d$ appears, with *d* some spatial distance and $k_{m,i}$ as defined in Eq. (51). Therefore, only a small number of distinct sine and cosine integrals has to be computed, which enables the efficient use of lookup tables. In the simulations performed in this work, the computation time for the trigonometric integrals could thus be reduced to a negligible fraction of the overall numerical cost.

The total computation time for the simulations presented in this work is on average in the range of a few hours for a single simulation (≈6 cores). When making use of routines to calculate the full matrix exponential in Eq. (54), the memory requirements are rather high since $N_{p}^{2}\times N_{x}$ values have to be stored. But, this is not necessary, of course. When employing a standard Runge–Kutta scheme only matrix–vector products of $D_{m,i,j}$ and the vector of cell averages $F_{i,j}(t)$ are needed. The use of lookup tables as described above enables the efficient calculation of the matrix elements $D_{m,i,j}$ on demand out of the numerically known values of $T^{F,v^{1}}(u)$ and $T^{\sigma ,v^{1}}(u)$, Eq. (49). With such a strategy the memory requirements reduce essentially to storing all entries of the Wigner function, which consists of $N_{p}\times N_{x}$ terms. Owing to the decoupling of the grid variables introduced by operator splitting, the method offers the direct possibility for massive parallelization. With this, a much higher number of grid points and even the simulation of two-dimensional problems is within reach.
